# Novel Echinacea formulations for the treatment of acute respiratory tract infections in adults—A randomized blinded controlled trial

**DOI:** 10.3389/fmed.2023.948787

**Published:** 2023-04-17

**Authors:** Johannes Sumer, Karin Keckeis, Giulia Scanferla, Manuel Frischknecht, Julia Notter, Ana Steffen, Philipp Kohler, Patrick Schmid, Bianca Roth, Kerstin Wissel, Pietro Vernazza, Peter Klein, Roland Schoop, Werner C. Albrich

**Affiliations:** ^1^Division of Infectious Diseases and Hospital Epidemiology, Cantonal Hospital St. Gallen, St. Gallen, Switzerland; ^2^Division of Infectious Diseases and Hospital Epidemiology, Cantonal Hospital of Lucerne, Luzern, Switzerland; ^3^Checkpoint Zürich, Zurich, Switzerland; ^4^d.s.h. Statistical Services GmbH, Rohrbach, Germany; ^5^A.Vogel AG, Roggwil, Switzerland

**Keywords:** *Echinacea purpurea*, randomized controlled trial, antiviral, respiratory tract infection, pharmaceutical formulations

## Abstract

**Background:**

*Echinacea purpurea* has clinical antiviral activity against respiratory viruses and modulates immune functions. In this study, we compared higher doses of new *Echinacea* formulations with conventional formulations at lower, preventive doses for therapy of respiratory tract infections (RTIs).

**Methods:**

In this randomized, blinded, controlled trial, healthy adults (*n* = 409) were randomized between November 2018 and January 2019 to one of four *Echinacea* formulations, which were taken in case of an RTI for up to 10 days. New formulations A (lozenges) and B (spray) delivered an increased dose of 16,800 mg/d *Echinacea* extract during days 1–3 and 2,240–3,360 mg/d afterward; as controls, conventional formulations C (tablets) and D (drops) delivered a lower daily dose of 2,400 mg, usually taken for prevention. The primary endpoint was time to clinical remission of first RTI episodes based on the Kaplan–Meier analysis of patient-reported, investigator-confirmed, respiratory symptoms assessed for up to 10 days. In a sensitivity analysis, the mean time to remission beyond day 10 was calculated by extrapolating the treatment effects observed on days 7 to 10.

**Results:**

A total of 246 participants (median age 32 years, 78% female participants) were treated for at least one RTI. Recovery by day 10 (complete absence of symptoms) was achieved in 56 and 44% of patients with the new and conventional formulations, respectively, showing a median time to recovery of 10 and 11 days, respectively (*p* = 0.10 in intention-to-treat analysis, *p* = 0.07 in per-protocol analysis). In the extrapolated sensitivity analysis, new formulations resulted in a significantly shorter mean time to remission (9.6 vs. 11.0 days, *p* < 0.001). Among those with an identified respiratory virus, viral clearance until day 10 based on real-time PCR from nasopharyngeal swabs was more frequent with new formulations (70 vs. 53%, *p* = 0.046). Tolerability and safety (adverse events: 12 vs. 6%, *p* = 0.19) were good and similar between formulations. There was one severe adverse event with a potential hypersensitivity reaction in a recipient of the novel spray formulation.

**Conclusion:**

In adults with acute RTI, new *Echinacea* formulations with higher doses resulted in faster viral clearance than conventional formulations in prophylactic dosages. The trend for faster clinical recovery was not significant by day 10 but became so upon extrapolation. A dose increase during acute respiratory symptoms might improve the clinical benefits of orally administered *Echinacea* formulations.

**Trial registration:**

The study was registered in the Swiss National Clinical Trials Portal (SNCTP000003069) and on ClinicalTrials.gov (NTC03812900; URL https://clinicaltrials.gov/ct2/show/NCT03812900?cond=echinacea&draw=3&rank=14).

## Introduction

Respiratory tract infections (RTIs) represent the most frequent illness in industrialized countries (1). The great majority of these infections are of viral origin (2), and over 200 respiratory pathogens have been identified. Despite being typically self-limiting (3), they are one of the leading causes of inappropriate antibiotic prescription and have a large societal impact (4, 5).

Effective therapies against influenza-like illness (ILI) or common cold are urgently needed, as currently available management is mainly symptomatic only (6). *Echinacea purpurea* was originally discovered as a medicinal plant by the indigenous population of North America who used it for the treatment of various ailments including respiratory tract infections, as an analgesic (tooth pain) as well as for snake bites (34). More recently, antiviral, anti-inflammatory, and immune-modulatory models of action were explored for particular plant preparations (7–9). It affects macrophage's antiviral function (10) and modulates chemokine and cytokine secretion (11). The virucidal activity is primarily directed against enveloped viruses such as influenza, parainfluenza, respiratory syncytial viruses (RSV), endemic coronaviruses, and SARS-CoV-2 (8, 11, 12). Randomized, controlled clinical studies (RCTs) have demonstrated efficacy for a 65% hydroethanolic extract from *Echinacea purpurea* herb and roots (Echinaforce^®^ [EF]) in the prevention and the treatment of RTIs including secondary complications (13–17). Five months of EF prevention during the COVID-19 pandemic (2020–2021) reduced enveloped virus infections by 43.2%, coronavirus infections by 48.3%, and SARS-CoV-2 by 63.1% (*p* < 0.05) (15). Moreover, Nicolussi et al. found significantly reduced enveloped virus infections in adults and children and >98% decreased viral loads with increased EF dosages used for the acute treatment (18).

In a previous clinical study on the treatment of the common cold, a preparation with a seven times higher content of *Echinacea* (16,800 mg EF daily) performed better than placebo and than dosages that are typically administered for the long-term prevention of RTIs (2,400 mg EF extract daily) (17).

We hypothesized that higher and more acute *Echinacea* doses increase therapeutic efficacy over preventively administered lower dosages. Furthermore, it is unknown whether liquid or solid formulations are more effective against different respiratory symptoms.

Therefore, we performed a randomized clinical trial (RCT) to investigate the superiority of higher doses of newly developed solid and liquid *Echinacea* formulations compared to preventive doses of conventional formulations regarding clinical efficacy, safety, and tolerability in the treatment of acute RTIs in healthy adults.

## Materials and methods

### Study design, participants, and randomization

This parallel-designed, blinded, RCT was conducted at the outpatient Clinic of Infectious Diseases and Hospital Epidemiology of the Cantonal Hospital St. Gallen. Healthy individuals of ≥18 years or those with respiratory symptoms of <24 h were invited to participate. Recruitment occurred through paper and electronic announcements throughout local hospitals and universities. Participants were enrolled by the study physicians from November 2018 to January 2019 after obtaining written informed consent. Inclusion and exclusion criteria are listed in Table 1. Baseline testing for hematological and metabolic parameters was performed.

**Table 1:** Inclusion and exclusion criteria.

**Inclusion criteria**	**Exclusion criteria**
Age ≥ 18 years Written informed consent Healthy or respiratory symptoms <24 h	Ongoing therapy with antimicrobial, antiviral, or immune-suppressive substances
	Atopy or bronchial asthma requiring medical therapy
	Bronchopulmonary dysplasia
	Chronic obstructive pulmonary disease
	Cystic fibrosis
	Immunological and degenerative disorders (autoimmune diseases, leukemia, lymphoma, myeloma, AIDS)
	Diabetes mellitus
	Chronic liver disease
	Chronic renal insufficiency
	Serious health conditions with reduced state of health or autoimmune or oncological illness
	Known allergies to plants of the *Compositae* family or to one of the excipients of the treatment
	Planned or recent surgery 3 months

Participants were randomly allocated by the study coordinator to receive either a conventional (drops or tablets, active control) or a newly developed *Echinacea* formulation (spray or lozenges) at a 1:1:1:1 ratio. A randomization list was generated by applying a block size of 8 using RANCODE, version 3.6 (IDV, Gauting, Germany) and retained by the study statistician (P.K.) in a sealed envelope. Participants received three sealed boxes of study medication allocated in ascending order according to the study medication number.

The formulations were fundamentally different with respect to taste, appearance, and method of application and were obviously distinguishable. Blinding of the study team and participants was nevertheless achieved by packing the medications into identically looking and labeled treatment boxes. Medications were handed out and returned exclusively in closed boxes in order to keep study personnel blind regarding the dispensed formulation. Likewise, participants were not instructed on the galenic appearance and posology of the respective new and conventional formulations.

At enrollment (visit 1), participants were instructed on symptoms of RTIs, and indications of when to start therapy with the assigned medication or when to immediately seek professional medical consultation. Study medication was issued to healthy subjects at inclusion for the prospective treatment of up to three future episodes of RTIs. Patients with acute RTI at inclusion, pre-existing for no more than 24 h, immediately started the first treatment. Concomitant medications for RTI episodes were allowed but recommended to be used only sparingly.

### Intervention and procedures

The participants were instructed to start treatment upon the occurrence of at least one respiratory symptom and/or the subjective feeling of a cold. They were told to inform the study team by phone to confirm treatment exclusion criteria (symptoms of complicated illness or pneumonia, in particular, confusion, dyspnea, neck pain or stiffness, thoracic chest pain, severe illness, or signs of non-respiratory tract infections). The participants were further instructed about the required diagnostic/monitoring procedures and received kits for self-collection of nasopharyngeal swabs (NPS) before and on days 5 and 9 of treatment of the first episode. Self-collection of the NPS was tutored at enrollment; an instruction brochure was provided, and a video was accessible on the study website. The kits included NPS (minitip Flocked Swabs, COPAN, Italy), vials with transport medium for storage at room temperature (Opti-Swab™, COPAN, Brescia, Italy), and sealable envelopes for postal delivery on the next working day to the laboratory (Labormedizinisches Zentrum Dr. Risch, Buchs, Switzerland) for semi-quantitative molecular detection of respiratory virus nucleic acid (Allplex^®^ RT-PCR, Seegene, South Korea) for 19 viruses (influenza A, influenza A H1, influenza A pdm09, influenza A H3, influenza B, respiratory syncytial virus (RSV) A, RSV B, parainfluenza virus (PIV) 1, PIV 2, PIV 3, PIV 4, human adenovirus, human enterovirus, human rhinovirus (A/B/C), human metapneumovirus, human bocavirus (1/2/3/4), coronavirus OC43, coronavirus 229E, and coronavirus NL63). Participants also received a diary to daily record the presence and severity of the symptoms of runny nose, congestion, sneezing, coughing, shivering, sore throat, malaise, headache, and myalgia using a Likert scale [*absent* (0), *mild* (1), *moderate* (2), or *severe* (3)], adverse events, and concomitant medications for each episode until recovery for up to 10 days.

During the first episode, participants were requested to return to the study center on day 5 (±1) for clinical evaluation, blood collection, verification of inclusion and exclusion criteria, diary entries, and medications (visit 2). Patients returned for a final visit (visit 3), which occurred between 3 weeks after starting the treatment for the first infection episode and 3 weeks after completion of the treatment for the third episode, depending on the last possible follow-up (5 July 2019).

### Treatments

All four investigated formulations (A–D) contained different concentrations of Echinaforce^®^ (EF) extract as the active substance, which is a 65% hydroethanolic extract made from freshly harvested *Echinacea purpurea* herb and roots at a ratio of 95:5 and uses a drug to extract ratio (DER) of 1:12 and 1:11. New formulations A (lozenges) and B (spray) delivered increased doses of 16,800 mg/d EF extract (7 × 2 puffs or five tablets) during days 1–3 and 2,240–3,360 mg/d (2–3 puffs or one tablet) afterward. Control formulations C (tablets) and D (drops) were used at 6 × 1 tablets or 6 × 10 drops, delivering a constant lower daily dose of 2,400 mg that is usually administered for prevention (Table 2). A typical characterization of the phytochemical marker substances found in EF extract is available in Sharma et al. (29). The formulations were filled into dark brown glass bottles sealed by screw closures and a nozzle (for the spray) and were manufactured and released by A. Vogel AG Switzerland under GMP guidelines. In the case of an RTI, the participants were instructed to use medications as described in Table 2. Formulations A to D were shown to contain 25 mg (A), 22 mg (B), 6.3 mg (C), and 4.6 mg (D) of dodeca-2E, 4E, 8E, and 10E/Z-tetraenoic acid isobutylamide per 100 g and contents remained stable throughout the duration of the study (>90% at 25 and 40°C). The stability of samples was further monitored by the TLC fingerprint analysis.

**Table 2:** Dosing of treatments: new Echinacea formulations (A and B) and conventional Echinacea formulations (C and D).

**Treatment group**	**Pharmaceutical form**	***Echinacea purpurea* extract per unit**	**Daily dose days 1–3**	**Daily dose days 4–10**
Formulation A	Lozenge	3,360 mg	5 × 1	1 × 1
Formulation B	Spray	1,200 mg	7 × 2	2–3 × 1
Formulation C	Tablets	400 mg	6 × 1	6 × 1
Formulation D	Drops	400 mg	6 × 1	6 × 1

Patient compliance was determined based on diary entries for each RTI episode and considered acceptable if the medication was taken on ≥75 % of days with symptoms of at least moderate severity. At the third visit, glass bottles were collected for pill count or weighing of solid or liquid drugs, respectively.

### Clinical outcomes

The primary endpoint was the duration until complete clinical remission of the first episode (all symptoms rated as *absent*) based on verified diary entries. The primary aim was to compare time to clinical remission for the new formulations A and B vs. conventional formulations C and D using the Kaplan–Meier analysis. Secondary outcomes included time to clinical remission if remission was defined as mild or absent symptoms, if all episodes were accounted for, cross-comparisons between different formulations, sick days defined by the presence of at least “*mild*” symptoms, and the patient's global impression of efficacy, safety, and tolerability rated as “*bad*,” “*moderate*,” “*good*,” or “*very good*” as a patient-centered outcome at the final study visit. Another secondary analysis of diary entries defined clinical remission as the time point when all symptoms in diaries were rated as “*mild”* or “*absent.”*

The virological response was defined as negative subsequent NPS (on day 5 or day 9/10) in individuals with a positive NPS on day 1 of symptoms. Virological non-responders were defined by a persistent positive test on day 9/10. There was no further viral testing beyond this date, i.e., no testing to confirm viral clearance. Subjectively perceived effectiveness and acceptance by the patient were compared between treatment groups as relative frequencies of Likert scores.

Cell counts, creatinine, transaminases, and bilirubin levels were measured at inclusion, on day 5 of the episode, and if abnormal at the final visit as safety endpoints. Adverse events and co-medications were compared between groups. All subjects who were administered at least one dose of study medication for an RTI represented the safety population.

The intention-to-treat (ITT) population consisted of all subjects with verified compliance based on returned medication glasses, who had at least one RTI episode, and who treated more than 75% of the episode days with at least “moderate” symptoms (i.e., no serious non-compliance). The per-protocol (PP) population consisted of all subjects in the ITT collective excluding those who had delayed follow-up (>28 days) and violated inclusion or exclusion criteria.

### Statistical analysis

Based on the results of previous studies (14, 19), a proportion of 98, 65, and 19% of symptomatic subjects was assumed for treatment days 2, 5, and 10 for new formulations (A and B) and of 99%, 83%, and 25% on the same treatment days for conventional formulations (C and D). From those frequencies, a median time to remission between days 6 and 7 (A and B) and between days 7 and 8 (C and D) could be expected. According to these assumptions, 127 patients were required for new (A and B) and conventional (C and D) formulation groups to reject equality between grouped treatments with an α error of 0.05 and 80% power (Gehan-Wilcoxon test). Due to uncertainty of how many participants would develop an RTI episode during the respiratory season, we decided to enroll at least 400 participants. The end of the study was defined as the time point when the first 300 RTI episodes were documented.

Statistical analyses were performed using SAS^®^ (version 9.4) and Testimate 6.5 (IDV, Data analysis and study planning, Gauting, Germany). For quantitative and ordinal variables, descriptive statistics were calculated; for categorical and dichotomous variables, absolute and relative numbers of score categories were determined. Clinical remission was reached when all symptoms were rated as absent (<1) and was analyzed using the Kaplan–Meier method and Peto log-rank test (assumption of late responses) or the Gehan-Wilcoxon test (assumption of early responses), as appropriate. The primary analysis compared new formulations (A and B) with conventional formulations (C and D) in the ITT population. Since there were only 223 instead of 300 targeted first episodes and since only ~50% of first episodes were fully resolved until the last day of documentation, group differences tended to be seen late (after day 5). Therefore, a sensitivity analysis extrapolated the mean slope of the recovery rate (between days 7 and 10) observed after day 10 until full recovery. Secondary efficacy endpoints were analyzed in an exploratory method using the Mantel–Haenszel chi-square test for ordinary variables, Fisher's exact test for dichotomous and categorical variables, and the *t*-test or analysis of variance (ANOVA) for quantitative variables. All statistical tests were performed two-sided at α = 0.05 significance level by a blinded statistician.

### Ethics and regulatory

The study was conducted in accordance with the latest version of the Declaration of Helsinki (October 2013) and ICH Good Clinical Practice (GCP) guidelines. Ethical approval was received by the local ethics committee (EKOS 2018-01383), and the trial was authorized by the Swiss national competent authority (Swissmedic 2018DR2140). The study was registered in the Swiss National Clinical Trials Portal (SNCTP000003069) and on ClinicalTrials.gov (NTC03812900).

## Results

**Figure 1:**
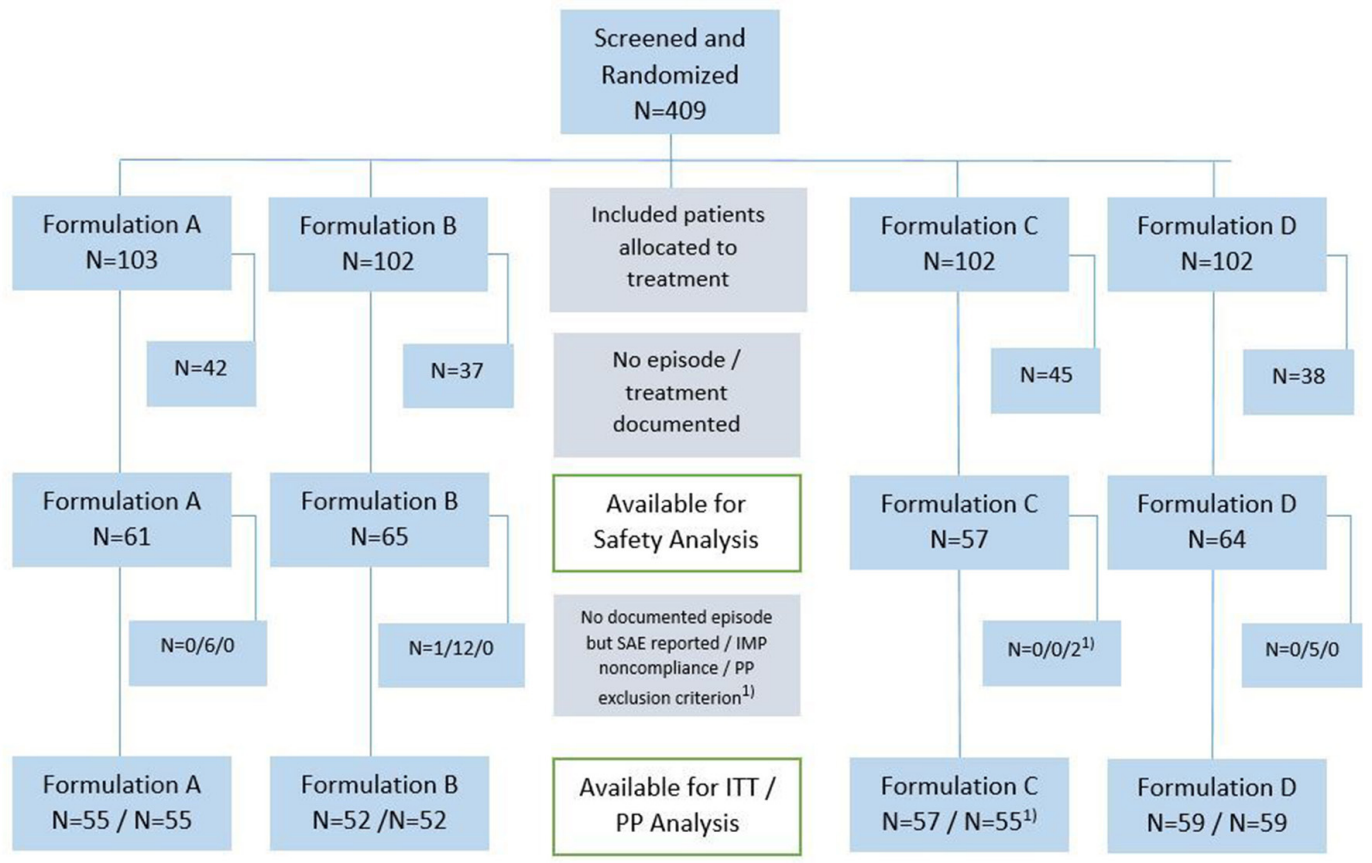
Study flow diagram. Number of patients are shown in both, PP and ITT populations; formulation A, lozenges; formulation B, spray; formulation C, conventional tablets; formulation D, conventional drops; RTI, respiratory tract infection; SAE, severe adverse event. ^1^Two patients were excluded from PP analysis due to seriously delayed follow-up phone call and medication of obstructive airways disease (exclusion criteria).

A total of 409 adults provided informed consent after screening and were randomized to one of the four treatment groups between November 2018 and January 2019 (Figure 1). The target of 400 participants was reached but, unexpectedly, they experienced only 246 RTIs by May 2019 (the end of the cold season), and the study was closed. The mean duration of observation of individual participants was 4.7 months (median 4.6 months) and more than 3 months in 80% of patients. A total of 11 patients prematurely terminated the study, one because of an adverse event and the others due to moving out of the area.

### Baseline characteristics

There was a similar sex distribution between groups (female: range 75–86%). The mean age (32 years) varied between groups (range 31.9–40.0 years). Otherwise, the four study arms were well-balanced for baseline characteristics (Table 3).

**Table 3:** Baseline characteristics.

**Parameter**	**Baseline Characteristics**					
	**Total (*****n*** = **223)**	**Echinacea A lozenges (*****n*** = **55)**	**Echinacea B spray (*****n*** = **52)**	**Echinacea C tablets (*****n*** = **57)**	**Echinacea D drops (*****n*** = **59)**	***P*** **-value** ^*^
Sex (females in %)	174 (78.0%)	47 (85.5%)	39 (75.0%)	43 (75.4%)	45 (76.3%)	0.479
Age—median (IQR)	32 (23.0–48.0)	30 (23.0–48.0)	38.5 (27.0–52.0)	27 (23.0–38.0)	36 (22.0–51.0)	0.04
BMI	22.7 (3.43)	22.4 (3.63)	22.9 (2.87)	21.8 (2.42)	23.5 (4.29)	0.083
Weight (kg)—mean (SD)	65.8 (12.83)	64.5 (12.28)	66.4 (12.94)	64.2 (11.18)	67.8 (14.62)	0.676
Height (cm)—mean (SD)	169.9 (8.63)	169.6 (7.47)	169.8 (9.19)	171.0 (9.49)	169.3 (8.38)	0.842
Relevant medical condition	54 (24.2%)	17 (30.9%)	15 (28.8%)	11 (19.3%)	11 (18.6%)	0.301^**^
**Comorbidities**						
Anemia	1 (0.4%)	0 (0.0%)	0 (0.0%)	1 (1.8%)	0 (0.0%)	
Hypothyroidism	5 (2.2%)	2 (3.6%)	2 (3.8%)	0 (0.0%)	1 (1.7%)	
Hypertension	8 (3.6%)	3 (5.5%)	3 (5.8%)	0 (0.0%)	2 (3.4%)	
Intake of Echinacea in last 3 months	32 (14.3%)	8 (14.5%)	8 (15.4%)	9 (15.8%)	7 (11.9%)	0.939
Mean number of infections per year (per participants' history)						0.403
0	6 (2.7%)	1 (1.8%)	1 (1.9%)	1 (1.8%)	3 (5.1%)	
1	90 (40.4%)	25 (45.5%)	22 (42.3%)	20 (35.1%)	23 (39.0%)	
2	83 (37.2%)	20 (36.4%)	19 (36.5%)	20 (35.1%)	24 (40.7%)	
3	35 (15.7%)	8 (14.5%)	8 (15.4%)	12 (21.1%)	7 (11.9%)	
4	6 (2.7%)	1 (1.8%)	1 (1.9%)	2 (3.5%)	2 (3.4%)	
5	1 (0.4%)	0 (0.0%)	0 (0.0%)	1 (1.8%)	0 (0.0%)	
6	2 (0.9%)	0 (0.0%)	1 (1.9%)	1 (1.8%)	0 (0.0%)	
Influenza vaccination						0.415
Yes	47 (21.1%)	12 (21.8%)	13 (25.0%)	15 (26.3%)	7 (11.9%)	
No	159 (71.3%)	40 (72.7%)	34 (65.4%)	37 (64.9%)	48 (81.4%)	
Unknown	17 (7.6%)	3 (5.5%)	5 (9.6%)	5 (8.8%)	4 (6.8%)	
Ongoing smoking	20 (9.0%)	3 (5.5%)	6 (11.5%)	5 (8.8%)	6 (10.2%)	0.719
**Co-medication at baseline**						
Corticosteroids/ glucocorticoids	2 (0.9%)	0 (0.0%)	0 (0.0%)	2 (3.6%)	0 (0.0%)	
Concomitant medications	72 (32.3%)	21 (38.2%)	17 (32.7%)	18 (31.6%)	16 (27.1%)	0.667^**^

* *P*-value using Kruskal–Wallis.

** Using Fisher's exact test.

### Primary endpoint

Most (246/409, 60.1%) participants were treated for at least one RTI, 31/246 (12.6%) for a second, and four (1.6%) for a third RTI episode. A total of 23 participants were excluded from the ITT and PP analyses mainly because of non-compliance with the intake of study medication, leaving 223 participants for the ITT analysis. Treatment compliance among the ITT population (i.e., treatment during >75% of days with moderate symptoms) was >98% in all study arms for the first episode.

**Figure 2:**
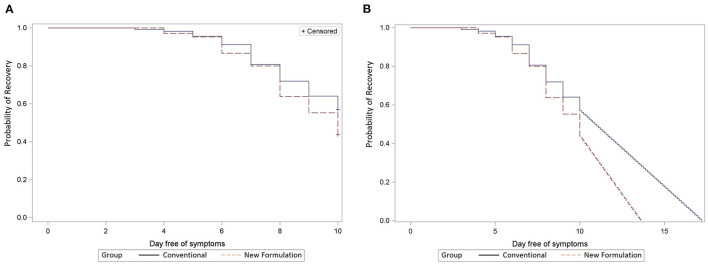
**(A)** Remission of first infection episode. Comparison of new (dashed line, A + B) vs. conventional (solid line, C + D) formulations in ITT population; x-axis: day free of symptoms; y-axis: the probability of recovery (Kaplan–Meier analysis). **(B)** Remission of first infection episode extrapolated until full recovery. Comparison of new (dashed line, A + B) versus conventional (solid line, C + D) formulations in ITT population; x-axis: day free of symptoms, y-axis: the probability of recovery (Kaplan–Meier analysis).

The median time to complete symptom recovery from the first episode as per RTI symptom assessment in diaries was not significantly different between the new and old formulations (10 and 11 days, *p* = 0.10 in ITT, *p* = 0.07 in PP; Figure 2A). Recovery curves diverged toward the end of treatment favoring the new formulations (A and B). Recovery was reached by day 10 in only 56% of participants with the new formulation and 44% with the conventional formulation. Extrapolating the recovery curve beyond day 10 presuming a linear continuation of recovery resulted in a 1.4-day reduction from 11.0 days with old formulations (C and D) to 9.6 days with new formulations (A and B; *p* < 0.001; Figure 2B).

### Secondary endpoints

In a secondary analysis of episode remission—predefined as RTI symptoms in diaries rated as “*absent*” or “*mild*”—there were no significant differences in time to remission between new and old formulations (median 6 vs. 7 days, *p* = 0.36).

**Figure 3:**
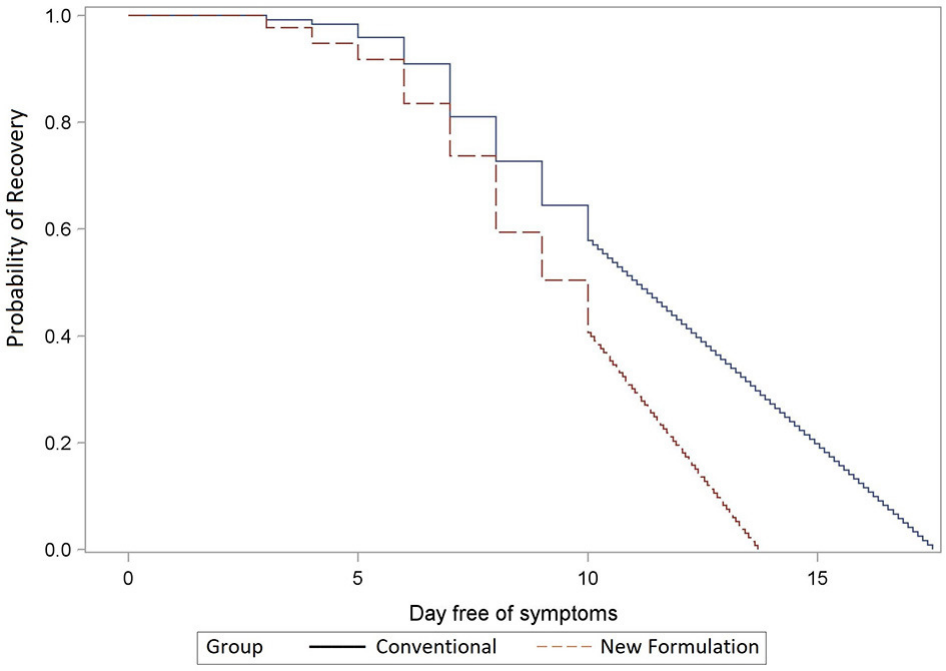
Remission of all infection episodes extrapolated until full recovery. Comparison of recovery times of new (dashed line, A + B) vs. conventional (solid line, C + D) (*p* < 0.001) formulations extrapolated beyond day 10 to absent symptoms; y-axis: the probability of recovery; x-axis: day free of symptoms; the Kaplan–Meier analysis.

Combining the effects on all episodes, including the 31 participants with second and the 4 with third episodes, the new formulations (A and B) resulted in faster recovery than conventional formulations (C and D; mean durations 8.5 vs. 9.0 days, median durations 10 vs. 11 days, *p* = 0.009). The mean difference of the extrapolated recovery times was 1.8 days (*p* <0 .001; Figure 3). For analyses of recovery to absent or mild symptoms, as used in previous publications (19, 20), there was a non-significant trend to faster recovery with new formulations (median 6d vs. 7d, *p* = 0.36 and median: 5d vs. 7d, *p* = 0.08, respectively; Supplementary Figure S1) when evaluating first and all treated episodes, respectively.

Several cross-comparisons were carried out in order to find differences between galenic forms (Supplementary Figure S2). Overall, there were no significant differences for any of these comparisons in the ITT population (*p* ranging from 0.07 to 0.6); however, extrapolated recovery times were significantly shorter for the new formulations than the conventional formulations (solid: 9.4d vs. 11.1d, *p* <0 .001; liquid: 10.1d vs. 10.9d, *p* = 0.03). Overall, there were no consistent or statistically significant differences between the different formulations regarding individual symptoms (Supplementary Figures S5a–i), except for slightly faster relief of sore throat with liquid preparations (reduction by up to 20% with new spray vs. conventional tablets on days 1–3) and faster responses of cough with new tablets (reduction by 42% on day 4 vs. conventional drops).

### Number of sick days

The mean number of sick days during the first episode was not different between solid formulations (4.7 d vs. 4.8 d; *p* = 0.74), but 4.6 and 5.0 sick days were found for the new spray and conventional drops, respectively (*p* = 0.46). The overall median number of sick days was 4 and 5 days for new and old formulations, respectively (*p* = 0.43).

### Global assessments

In total, 53% of participants (*n* = 57/107) correctly judged that they received a new formulation, and 46% judged correctly that they received an old formulation (*n* = 54/116, Supplementary Table S1).

The participants' global assessment of treatment effectiveness at the study end was better with new than with conventional formulation for the first episode (62.5 vs. 48.2% ratings “*good*” or “*very good*,” *p* = 0.02) and was more pronounced for the liquid (64.7 vs. 46.5%) than the solid (60.3 vs. 50.0%) formulations.

### RTI complications and antibiotic use

Of 288 RTI with *Echinacea*-treated episodes, three required antibiotic therapy. Excluding one patient who received antibiotics from the start for sinusitis, the remaining two patients (0.7%) who required subsequent treatment with antibiotics for tonsillitis and a non-specified bacterial infection, respectively, had received conventional Echinacea drops (2/64, 3.1%).

### Virus identification

The nasopharyngeal specimen analyses most commonly detected rhinovirus (28.7%) and endemic coronaviruses (23.8%) (Supplementary Table S2). Virological responders were more frequent with the new (*n* = 48/69) than the conventional formulations (*n* = 44/83 or 70 vs. 53%, *p* = 0.046). This difference was mainly due to the effects of the new lozenges (*n* = 28/35 or 80%) in comparison to 58.8% (*n* = 20/34) with conventional tablets (*p* = 0.03, Supplementary Table S3).

### Adverse events

Overall, 21 patients experienced AEs with the new formulations (16.6%) and 22 patients with the old formulations (18.0%), showing no difference (*p* = 0.87). In 22 patients, events were assumed adverse drug reactions (ADRs), and two of them were classified as serious: one with a potential link to the novel spray formulation (hypersensitivity reaction; Table 4 and Supplementary Table S4). Despite assessment by allergy specialists including oral and epicutaneous re-challenge assays, it remained unclear whether this was caused by *Echinacea* or the excipients used, such as anise oil, which is known to trigger such reactions (21).

**Table 4:** Adverse events.

		**Echinacea A lozenge (*N* = 61)**	**Echinacea B spray (*N* = 66)**	**Echinacea C tablet (*N* = 57)**	**Echinacea D drops (*N* = 64)**
		***N*** **(%)**	***N*** **(%)**	***N*** **(%)**	***N*** **(%)**
Patients with	Adverse events	10 (16.4%)	11 (16.7%)	8 (14.0%)	14 (21.9%)
	Drug related AEs^a^	9 (14.8%)	6 (9.1%)	3 (5.3%)	4 (6.3%)
	Serious AEs	0 (0.0%)	2 (3.0%)	0 (0.0%)	0 (0.0%)
	Drug related serious adverse events^a^	0 (0.0%)	1 (1.5%)	0 (0.0%)	0 (0.0%)

Adverse events in the safety population.

a Causality judged at least possible.

Tolerability was rated better in the group with conventional than with novel formulations (98.3 vs. 87.6% with good or very good, *p* = 0.007).

There was no apparent difference in the use of co-medications between treatment groups (Supplementary Table S5).

### Blood laboratory evaluation

Statistically significant changes in some laboratory values on day 5 (±1 day) after treatment initiation for the first episode in comparison to the inclusion visit (decreases in absolute lymphocyte counts) were observed but those were small, remained within normal limits, and were judged as related to the infectious episodes. All initially abnormal values returned to normal levels at the final visit. No consistent differences were observed between different *Echinacea* formulations.

## Discussion

This study showed possible beneficial effects of new Echinacea formulations used at higher cumulative doses compared to conventional formulations (in prophylactic dosages) for acute RTI treatment in adults. First, the new formulations resulted in a significantly shorter time to complete recovery using an extrapolated analysis and an improved subjective response rate. Second, new formulations had a greater antiviral efficacy with faster viral clearance. Otherwise, there were only minor differences between the new liquid and solid formulations and no significant difference at day 10 of the first episode at a generally low rate of antibiotic prescription.

The tested new formulations reduced time to recovery by 1–1.4 days, which is similar to the reduction in symptom duration for the common cold by 1.4 days compared to placebo in a large meta-analysis (22). Comparison of our results with previous data is hampered by the variety of different *Echinacea* products and formulations available worldwide which vary in plant species and parts used, harvesting region and season, extraction methods, formulation, and dosages (23). Therefore, each preparation has to be evaluated individually or at least grouped by similar formulations. Generally, lipophilic extracts were shown to have a higher efficacy than pressed juices in the prevention of recurrent infections (16). Dose-dependent effects as seen in our study were previously also demonstrated in two studies using the same EF extract (17, 19). A recent treatment study in children showed that a dose of 2,000 mg EF extract reduced the duration of cold episodes by 1.7 days compared to a 1,200 mg dose (19). A dose seven times higher than the standard dose (16,800 vs. 2,400 mg/d) in adults achieved a 63 vs. a 45% reduction of symptoms after 5–7 days in a placebo-controlled randomized trial (17). Overall, all available studies provide evidence of increased therapeutic benefits for increased dosages over lower dosages, usually applied for the prevention of RTIs.

There were two main reasons for our study being underpowered to show a difference between formulations. First, our power analysis assumed faster symptom resolution than observed in our study (3, 19, 20, 24). Only half of the patients achieved a complete recovery by day 10, and therefore, there were fewer recovery events for the Kaplan–Meier analysis. In contrast, Raus et al. (20) demonstrated a comparable effect between Echinaforce^®^ Hot Drink and oseltamivir in patients with influenza-like illness after 10 days of treatment reaching symptom alleviation (rated as mild or absent) in 90 vs. 84.8%. Similarly, complete symptom resolution was achieved in 90% of children by day 10 (19). It is unclear why symptom duration was longer in our participants, who all received *Echinacea* preparations. Since many participants in our study were healthcare workers, they might have particularly diligently documented their symptoms and rated very minor symptoms as present, leading to delayed resolution. We defined the resolution of symptoms as the complete absence of respiratory symptoms, which might be considered clinically less relevant and was stricter than in those studies mentioned earlier (19, 20). Second, participants experienced fewer RTI episodes than expected possibly due to a mild influenza season in 2018/2019 (33). As such, this study was unable to falsify the null hypothesis of no benefit of newly developed over conventional EF formulations.

In an attempt to increase statistical power, missing data points were substituted through extrapolation of recovery trends observed on days 7–10 to illustrate the entire course of the disease beyond day 10. These measures allowed a more detailed assessment of symptom resolution, indicating a significant difference by 1.4 days, which is similar to the benefit of oseltamivir in influenza and is clinically relevant (25).

There was no consistent difference between liquid or solid formulations. The trend for a faster resolution of nasal complaints and sore throat (data not shown) with the new spray might be due to the local anti-inflammatory and immunomodulatory effect of alkylamides, which are considered the main active principle of Echinacea (26, 27).

*In vitro*, EF extract has antiviral activity, especially against enveloped viruses including influenza, parainfluenza viruses, coronaviruses (including SARS-CoV-2), or respiratory syncytial virus, and considerably less antiviral activity against non-enveloped viruses such as rhinovirus and adenovirus (12, 28, 29). Interestingly, the strongest antiviral effects were found when epithelial cells were in direct contact with EF indicating immediate treatment or prophylactic benefits rather than with post-exposure of cells (8, 12). In randomized, double-blind placebo-controlled trials, the prophylactic use of EF significantly reduced the incidence of laboratory-confirmed viral RTIs and was non-inferior to the placebo in terms of safety (13–15). Similar results were found for Echinaforce Hot Drink^®^ which was non-inferior to oseltamivir in the treatment of ILI.

Vos et al. (3) showed that the baseline viral load was associated with symptom severity in RTIs but not with symptom duration. In our study, significantly more patients turned from initial positive detections to negative RT-PCR results for any virus detected using “new” galenic with considerably higher EF doses. Remarkably, the rate of antibiotic intake was very low in both groups compared to often inappropriate high prescriptions in RTI. Echinacea in both dosages could have contributed to this finding.

The new formulations were slightly less well-tolerated than conventional formulations. The majority of adverse events were related to the known and expected “tingling” sensation of alkylamides in the more highly concentrated new formulations. One severe adverse event (hypersensitivity reaction) was documented, in which a causal link could not be excluded. There were no safety differences between formulations with regard to laboratory parameters. Overall, adverse events were rare in the present study and confirmed the known good safety profile for *Echinacea* (13, 17, 20, 30, 31).

A limitation was the inability to perform complete blinding as formulations were different in terms of application. However, reasonably good blinding was achieved in that approximately only half of participants correctly judged their treatment group assignment to “new” or “conventional” *Echinacea* formulations, while approximately a third were unclear, and 10–20% of participants wrongly guessed the medication's identity. The lack of a placebo arm is a weakness of this study, but earlier studies showed the benefit of *Echinacea* preparations over placebo in common colds (17) or similar efficacy compared to oseltamivir in influenza (20). Furthermore, blinding in a placebo-controlled study would be difficult to achieve due to the tingling sensation with EF. On the contrary, the effect of the intervention group (new formulations) was measured against an active control of conventional EF in prophylactic dosages rather than an inactive placebo.

The usage of prophylactically dosed *Echinacea* formulations in the control group might have influenced the results. Possibly, the usage of higher therapeutic dosages might have resulted in less or not even any difference compared to the new formulations.

The strengths of this study are the randomized controlled trial design with systematic daily documentation of symptoms in the diary, which was confirmed by the study team. In addition, viral testing using semi-quantitative multiplex PCR allowed us to determine an antiviral response. A high proportion of the study subjects were healthcare workers with a high sensitivity and motivation to report even residual symptoms that others might have considered resolved. Thus, the population is representative of generally healthy younger adults. The professional qualification might have also supported the quality of the nasopharyngeal swab collection. In addition, it has been recently shown that instructed anterior nasal self-sampling had similar sensitivities to professional nasopharyngeal for viral PCR (32).

## Conclusion

In adults with acute RTI, new *Echinacea* formulations at higher initial doses resulted in a faster viral clearance than conventional formulations at prophylactic dosages. The trend for a faster clinical recovery was not significant by day 10 but became so upon extrapolation. A dose increase during acute respiratory symptoms might improve the clinical benefits of orally administered EF formulations.

## Data availability statement

The raw data supporting the conclusions of this article will be made available by the authors, without undue reservation.

## Ethics statement

The studies involving human participants were reviewed and approved by the Local Ethics Committee (EKOS 2018-01383). The patients/participants provided their written informed consent to participate in this study.

## Author contributions

The study was conceptualized by WA, KK, JS, and RS, with the help of all co-authors. Patients were recruited by WA, KK, JS, GS, MF, JN, AS, PKo, PKl, BR, KW, and PS. WA, KK, and JS carried out the study, collected study data, and were involved in data interpretation. KK and JS drafted the first version of the manuscript. PKl carried out data management and statistical analysis. PKl and RS assisted in the data interpretation and writing of the manuscript. All authors contributed to the final version of the manuscript.

## References

[1] MontoAS. Epidemiology of viral respiratory infections. Am J Med. (2002) 112(Suppl. 6A):4S−12. 10.1016/S0002-9343(01)01058-011955454

[2] Mäkelä MJ Puhakka T Ruuskanen O Leinonen M Saikku P Kimpimäki M. Viruses and bacteria in the etiology of the common cold. J Clin Microbiol. (1998) 36:539–42. 10.1128/JCM.36.2.539-542.1998 9466772 PMC104573

[3] Vos LM Bruyndonckx R Zuithoff NPA Little P Oosterheert JJ Broekhuizen BDL. Lower respiratory tract infection in the community: associations between viral aetiology and illness course. Clin Microbiol Infect. (2021) 27:96–104. 10.1016/j.cmi.2020.03.023 32244051 PMC7118666

[4] Antibiotic Use for Viral Acute Respiratory Tract Infections Remains Common. AJMC. Available online at: https://www.ajmc.com/view/antibiotic-use-for-viral-acute-respiratory-tract-infections-remains-common (accessed September 18, 2020). 26619058

[5] Goossens H Ferech M Vander Stichele R Elseviers M ESAC Project Group. Outpatient antibiotic use in Europe and association with resistance: a cross-national database study. Lancet Lond Engl. (2005) 365:579–87. 10.1016/S0140-6736(05)17907-0 15708101

[6] Kim SY Chang Y Cho HM Hwang Y Moon YS. Non-steroidal anti-inflammatory drugs for the common cold. Cochrane Database Syst Rev. (2015) 2015:CD006362. 10.1002/14651858.CD006362.pub4 23733384

[7] Sharifi-Rad M Mnayer D Morais-Braga MFB Carneiro JNP Bezerra CF Coutinho HDM. Echinacea plants as antioxidant and antibacterial agents: from traditional medicine to biotechnological applications. Phytother Res PTR. (2018) 32:1653–63. 10.1002/ptr.6101 29749084

[8] Pleschka S Stein M Schoop R Hudson JB. Anti-viral properties and mode of action of standardized Echinacea purpurea extract against highly pathogenic avian influenza virus (H5N1, H7N7) and swine-origin H1N1 (S-OIV). Virol J. (2009) 6:197. 10.1186/1743-422X-6-197 19912623 PMC2785784

[9] Sharma SM Anderson M Schoop SR Hudson JB. Bactericidal and anti-inflammatory properties of a standardized Echinacea extract (Echinaforce): dual actions against respiratory bacteria. Phytomedicine Int J Phytother Phytopharm. (2010) 17:563–8. 10.1016/j.phymed.2009.10.022 20036523

[10] Senchina DS Martin AE Buss JE Kohut ML. Effects of Echinacea extracts on macrophage antiviral activities. Phytother Res PTR. (2010) 24:810–6. 10.1002/ptr.2991 20041425

[11] Sharma M Arnason JT Burt A Hudson JB. Echinacea extracts modulate the pattern of chemokine and cytokine secretion in rhinovirus-infected and uninfected epithelial cells. Phytother Res PTR. (2006) 20:147–52. 10.1002/ptr.1824 16444669

[12] Signer J Jonsdottir HR Albrich WC Strasser M Züst R Ryter S. *In vitro* virucidal activity of Echinaforce^®^, an Echinacea purpurea preparation, against coronaviruses, including common cold coronavirus 229E and SARS-CoV-2. Virol J. (2020) 17:136. 10.1186/s12985-020-01401-2 32907596 PMC7479405

[13] Jawad M Schoop R Suter A Klein P Eccles R. Safety and efficacy profile of *Echinacea purpurea* to prevent common cold episodes: a randomized, double-blind, placebo-controlled trial. Evid Based Complement Alternat Med. (2012) 2012:1–7. 10.1155/2012/841315 23024696 PMC3457740

[14] Ogal M Johnston SL Klein P Schoop R. Echinacea reduces antibiotic usage in children through respiratory tract infection prevention: a randomized, blinded, controlled clinical trial. Eur J Med Res. (2021) 26:33. 10.1186/s40001-021-00499-6 33832544 PMC8028575

[15] Kolev E Mircheva L Edwards MR Johnston SL Kalinov K Stange R. Echinacea purpurea for the long-term prevention of viral respiratory tract infections during COVID-19 pandemic: a randomized, open, controlled, exploratory clinical study. Front Pharmacol. (2022) 13:856410. 10.3389/fphar.2022.856410 35559249 PMC9087554

[16] Schapowal A Klein P Johnston SL. Echinacea reduces the risk of recurrent respiratory tract infections and complications: a meta-analysis of randomized controlled trials. Adv Ther. (2015) 32:187–200. 10.1007/s12325-015-0194-4 25784510

[17] Brinkebron RM Shah DV Degenring FH. Echinaforce and other Echinacea fresh plant preparations in the treatment of the common cold. A randomized, placebo controlled, double-blind clinical trial. Phytomedicine. (1999) 6:1–6. 10.1016/S0944-7113(99)80027-0 10228604

[18] Nicolussi S Ardjomand-Woelkart K Stange R Gancitano G Klein P Ogal M. Echinacea as a potential force against coronavirus infections? A mini-review of randomized controlled trials in adults and children. Microorganisms. (2022) 10:211. 10.3390/microorganisms10020211 35208665 PMC8879308

[19] Weishaupt R Bächler A Feldhaus S Lang G Klein P Schoop R. Safety and dose-dependent effects of echinacea for the treatment of acute cold episodes in children: a multicenter, randomized, open-label clinical trial. Child Basel Switz. (2020) 7:E292. 10.3390/children7120292 33333722 PMC7765151

[20] Raus K Pleschka S Klein P Schoop P Fischer P. Effect of an Echinacea-based hot drink versus oseltamivir in influenza treatment: a randomized, double-blind, double-dummy, multicenter, noninferiority clinical trial. Curr Ther Res Clin Exp. (2015) 77:66–72. 10.1016/j.curtheres.2015.04.001 26265958 PMC4528044

[21] Jensen-Jarolim E Leitner A Hirschwehr R Kraft D. Characterization of allergens in Apiaceae spices: anise, fennel, coriander and cumin. Clin Exp Allergy J Br Soc Allergy Clin Immunol. (1997) 27:1299–306. 10.1046/j.1365-2222.1997.1580956.x 9420134

[22] Shah SA Sander S White CM Rinaldi M Coleman CI. Evaluation of echinacea for the prevention and treatment of the common cold: a meta-analysis. Lancet Infect Dis. (2007) 7:473–80. 10.1016/S1473-3099(07)70160-3 17597571 PMC7106401

[23] Barrett B. Medicinal properties of Echinacea: a critical review. Phytomed Int J Phytother Phytopharm. (2003) 10:66–86. 10.1078/094471103321648692 12622467

[24] Schulten B Bulitta M Ballering-Brühl B Köster U Schäfer M. Efficacy of Echinacea purpurea in patients with a common cold. A placebo-controlled, randomised, double-blind clinical trial. Arzneimittelforschung. (2001) 51:563–8. 10.1055/s-0031-1300080 11505787

[25] Fry AM Goswami D Nahar K Sharmin AT Rahman M Gubareva L. Efficacy of oseltamivir treatment started within 5 days of symptom onset to reduce influenza illness duration and virus shedding in an urban setting in Bangladesh: a randomised placebo-controlled trial. Lancet Infect Dis. (2014) 14:109–18. 10.1016/S1473-3099(13)70267-6 24268590

[26] Woelkart K Dittrich P Beubler E Pinl F Schoop R Suter A. Pharmacokinetics of the main alkamides after administration of three different *Echinacea purpurea* preparations in humans. Planta Med. (2008) 74:651–6. 10.1055/s-2008-1034284 18240099

[27] Woelkart K Bauer R. The role of alkamides as an active principle of echinacea. Planta Med. (2007) 73:615–23. 10.1055/s-2007-981531 17538868

[28] Hudson J Vimalanathan S. Echinacea—a source of potent antivirals for respiratory virus infections. Pharmaceuticals. (2011) 4:1019–31. 10.3390/ph4071019

[29] Sharma M Anderson SA Schoop R Hudson JB. Induction of multiple pro-inflammatory cytokines by respiratory viruses and reversal by standardized Echinacea, a potent antiviral herbal extract. Antiviral Res. (2009) 83:165–70. 10.1016/j.antiviral.2009.04.009 19409931

[30] David S Cunningham R. Echinacea for the prevention and treatment of upper respiratory tract infections: a systematic review and meta-analysis. Complement Ther Med. (2019) 44:18–26. 10.1016/j.ctim.2019.03.011 31126553

[31] Ross SM. Echinacea purpurea: a proprietary extract of echinacea purpurea is shown to be safe and effective in the prevention of the common cold. Holist Nurs Pract. (2016) 30:54–7. 10.1097/HNP.0000000000000130 26633727

[32] Lindner AK Nikolai O Kausch F Wintel M Hommes F Gertler M. Head-to-head comparison of SARS-CoV-2 antigen-detecting rapid test with self-collected nasal swab versus professional-collected nasopharyngeal swab. Eur Respir J. (2021) 57:2003961. 10.1183/13993003.03961-2020 33303544 PMC7736752

[33] BAG Bulletin 29/2019 Switzerland Issue 15. (2019). p. 9–20

[34] Bauer R Wagner W. Echinacea. Ein Handbuch für Ärzte, Apotheker und andere Naturwissenschaftler. Stuttgart: Wissenschaftliche Verlagsgesellschaft (1990).

