# Supplementary material for: Novel Echinacea formulations for the treatment of acute respiratory tract infections in adults—A randomized blinded controlled trial
Source: Front Med (Lausanne). 2023 Apr 17;10:948787. doi: 10.3389/fmed.2023.948787 (PMC10150997; doi:10.3389/fmed.2023.948787)

Supplementary Material

**1.1 Supplemental tables**

Table S1: Judgement about identification of medication used – Blinding effectiveness

|  | Echinacea A  lozenge. (n = 55) | Echinacea B spray (n = 52) | Echinacea C  tablet (n = 57) | Echinacea D  drops (n = 59) |
| --- | --- | --- | --- | --- |
| Guess | N (%) | N (%) | N (%) | N (%) |
| New | 29 (52.7%) | 28 (53.8%) | 6 (10.5%) | 14 (23.7%) |
| Conventional | 7 (12.7%) | 4 (7.7%) | 32 (56.1%) | 22 (37.3%) |
| Not known | 17 (30.9%) | 18 (34.6%) | 19 (33.3%) | 20 (33.9%) |
| Not applicable or missing | 2 (3.6%) | 2 (3.8%) | 0 (0.0%) | 3 (5.1%) |
| Total | 55 (100.0%) | 52 (100.0%) | 57 (100.0%) | 59 (100.0%) |
| New | 57 (53.3%) | | 20 (17.2%) | |
| Conventional | 11 (10.3%) | | 54 (46.6%) | |
| Not known | 35 (32.7%) | | 39 (33.6%) | |
| Not applicable or missing | 4 (3.7%) | | 3 (2.6%) | |
| Total | 107 (100.0%) | | 116 (100.0%) | |

Table S2: Virus Identification

|  | Echinacea A lozenge (N = 55) | Echinacea B spray (N = 52) | Echinacea C Tablet (N = 57) | Echinacea D drops (N = 59) | Total (N = 223) |
| --- | --- | --- | --- | --- | --- |
| Agent* | N (%) | N (%) | N (%) | N (%) | N (%) |
| **Rhinovirus** | **11 (20.0%)** | **15 (28.8%)** | **21 (36.8%)** | **17 (28.8%)** | **64 (28.7%)** |
| **Influenza** | **4 (7.3%)** | **5 (9.6%)** | **1 (1.8%)** | **5 (8.5%)** | **15 (6.7%)** |
| Flu_A | 4 (7.3%) | 4 (7.7%) | 1 (1.8%) | 3 (5.1%) | 12 (5.4%) |
| H3 | 1 (1.8%) | 3 (5.8%) | 0 (0.0%) | 3 (5.1%) | 7 (3.1%) |
| pdm09 | 3 (5.5%) | 2 (3.8%) | 1 (1.8%) | 1 (1.7%) | 7 (3.1%) |
| **Parainfluenza** | **1 (1.8%)** | **1 (1.9%)** | **1 (1.8%)** | **2 (3.4%)** | **5 (2.2%)** |
| PIV1 | 0 (0.0%) | 0 (0.0%) | 1 (1.8%) | 0 (0.0%) | 1 (0.4%) |
| PIV3 | 0 (0.0%) | 0 (0.0%) | 0 (0.0%) | 2 (3.4%) | 2 (0.9%) |
| PIV4 | 1 (1.8%) | 1 (1.9%) | 0 (0.0%) | 0 (0.0%) | 2 (0.9%) |
| **Respiratory syncytial virus (RSV)** | **4 (7.3%)** | **2 (3.8%)** | **4 (7.0%)** | **8 (13.6%)** | **18 (8.1%)** |
| RSV_A | 1 (1.8%) | 1 (1.9%) | 0 (0.0%) | 2 (3.4%) | 4 (1.8%) |
| RSV_B | 3 (5.5%) | 1 (1.9%) | 4 (7.0%) | 6 (10.2%) | 14 (6.3%) |
| **Coronavirus** | **17 (30.9%)** | **10 (19.2%)** | **19 (33.3%)** | **7 (11.9%)** | **53 (23.8%)** |
| NL63 | 6 (10.9%) | 2 (3.8%) | 8 (14.0%) | 3 (5.1%) | 19 (8.5%) |
| OC43 | 5 (9.1%) | 5 (9.6%) | 2 (3.5%) | 2 (3.4%) | 14 (6.3%) |
| _229E | 6 (10.9%) | 3 (5.8%) | 9 (15.8%) | 2 (3.4%) | 20 (9.0%) |
| **Metapneumovirus (MPV)** | **0 (0.0%)** | **1 (1.9%)** | **1 (1.8%)** | **0 (0.0%)** | **2 (0.9%)** |
| **Bocavirus** | **0 (0.0%)** | **1 (1.9%)** | **0 (0.0%)** | **1 (1.7%)** | **2 (0.9%)** |
| **Adenovirus** | **0 (0.0%)** | **0 (0.0%)** | **0 (0.0%)** | **1 (1.7%)** | **1 (0.4%)** |

* more than one agent could be detected per patient

Table S3: Virological response

|  | New formulation (N = 107) | Conventional formulation (N = 116) | Echinacea A lozenge (N = 55) | Echinacea C tablet (N = 57) | Echinacea B  spray (N = 52) | Echinacea D drops (N = 59) |
| --- | --- | --- | --- | --- | --- | --- |
| Responder | N (%) | N (%) | N (%) | N (%) | N (%) | N (%) |
| Missing | 39 | 33 | 21 | 13 | 18 | 20 |
| No | 21 (30.4%) | 39 (47.0%) | 7 (20.0%) | 20 (45.5%) | 14 (41.2%) | 19 (48.7%) |
| Yes | 48 (69.6%) | 44 (53.0%) | 28 (80.0%) | 24 (54.5%) | 20 (58.8%) | 20 (51.3%) |
| Fisher's test | P = 0.046 | | P = 0.031 | | P = 0.638 | |

Intention-to treat population; response was judged for any virus identified

Table S4: Drug related adverse events by MedDRA Primary System Organ Class and Preferred Term - Safety population

| **System Organ Class** | Echinacea A lozenge (N = 9) | Echinacea B 1200 spray (N = 6) | Echinacea C tablet (N = 4) | Echinacea D drops (N = 4) |
| --- | --- | --- | --- | --- |
| Preferred Term | N (%) | N (%) | N (%) | N (%) |
| **General diseases and complaints at site of administration** | 1 (11.1%) | 2 (33.3%) | 0 (0.0%) | 0 (0.0%) |
| Fatigue | 0 (0.0%) | 1 (16.7%) | 0 (0.0%) | 0 (0.0%) |
| Pain at site of application | 0 (0.0%) | 1 (16.7%) ^1)^ | 0 (0.0%) | 0 (0.0%) |
| Feeling unwell | 1 (11.1%) | 0 (0.0%) | 0 (0.0%) | 0 (0.0%) |
| **Eye diseases** | 2 (22.2%) | 0 (0.0%) | 0 (0.0%) | 0 (0.0%) |
| Swelling of the eyelid | 2 (22.2%) | 0 (0.0%) | 0 (0.0%) | 0 (0.0%) |
| **Diseases of the skin and hypoderm** | 0 (0.0%) | 0 (0.0%) | 0 (0.0%) | 1 (25.0%) |
| Rash | 0 (0.0%) | 0 (0.0%) | 0 (0.0%) | 1 (25.0%) |
| **Diseases of the blood and lymph system** | 0 (0.0%) | 0 (0.0%) | 1 (25.0%) | 0 (0.0%) |
| **Diseases of the gastrointestinal tract** | 6 (66.7%) | 4 (66.7%) | 3 (75.0%) | 3 (75.0%) |
| Abdominal complaints | 2 (22.2%) | 0 (0.0%) | 0 (0.0%) | 0 (0.0%) |
| Abdominal pain | 0 (0.0%) | 0 (0.0%) | 1 (1.8%) | 0 (0.0%) |
| Anaesthesia oral | 1 (11.1%) ^1)^ | 0 (0.0%) | 0 (0.0%) | 0 (0.0%) |
| Feeling of sickness | 0 (0.0%) | 2 (33.3%) ^1)^ | 0 (0.0%) | 0 (0.0%) |
| Diarrhea | 0 (0.0%) | 1 (16.7%) | 1 (25.0%) | 2 (50.0%) |
| Dyspepsia | 1 (11.1%) | 0 (0.0%) | 1 (25.0%) | 1 (25.0%) |
| Vomitus | 0 (0.0%) | 2 (33.3%) ^1)^ | 0 (0.0%) | 0 (0.0%) |
| Hypoaesthesia oral | 1 (11.1%) ^1)^ | 0 (0.0%) | 0 (0.0%) | 0 (0.0%) |
| Oral complaints | 1 (11.1%) ^1)^ | 0 (0.0%) | 0 (0.0%) | 0 (0.0%) |
| Paraesthesia oral | 1 (11.1%) ^1)^ | 0 (0.0%) | 0 (0.0%) | 0 (0.0%) |
| Nausea | 2 (22.2%) ^1)^ | 1 (16.7%) ^1)^ | 1 25.0%) | 0 (0.0%) |
| **Diseases of the immune system** | 0 (0.0%) | 1 (16.7%) | 0 (0.0%) | 0 (0.0%) |
| Hypersensitivity | 0 (0.0%) | 1 (16.7%) | 0 (0.0%) | 0 (0.0%) |
| **Diseases of the nerve system** | 2 (22.2%) | 2 (33.3%) | 0 (0.0%) | 0 (0.0%) |
| Dysgeusia | 1 (11.1%) ^1)^ | 0 (0.0%) | 0 (0.0%) | 0 (0.0%) |
| Disturbed taste | 1 (11.1%) ^1)^ | 2 (33.3%) ^1)^ | 0 (0.0%) | 0 (0.0%) |
| Paraesthesia | 1 (11.1%) ^1)^ | 0 (0.0%) | 0 (0.0%) | 0 (0.0%) |
| Somnolence | 0 (0.0%) | 1 (16.7%) | 0 (0.0%) | 0 (0.0%) |

^1)^ Potentially associated with the new galenic form, i.e. tingling sensation or the subjective aversion against the formulation/taste of tablets and spray.

Table S5: Concomitant medication or therapies by ATC 4 level– Safety population

|  | Echinacea A  3360 mg Tbl. (N = 61) | Echinacea B  1200 mg Sprühst. (N = 66) | Echinacea C 400 mg Tbl. (N = 57) | Echinacea D 400 mg Tropf. (N = 64) |
| --- | --- | --- | --- | --- |
| Medication or therapies | N (%) | N (%) | N (%) | N (%) |
| **Medication stopped before V1** |  |  |  |  |
| ..BETA BLOCKINGAGENTS | 0 ( 0.0%) | 1 ( 1.5%) | 0 ( 0.0%) | 0 ( 0.0%) |
| ..GENERAL | 1 ( 1.6%) | 2 ( 3.0%) | 0 ( 0.0%) | 2 ( 3.1%) |
| NUTRIENTS |  |  |  |  |
| ..HORMONAL | 0 ( 0.0%) | 0 ( 0.0%) | 1 ( 1.8%) | 0 ( 0.0%) |
| CONTRACEPTIVES FOR SYSTEMIC USE |  |  |  |  |
|  |  |  |  |  |
| ..IRON PREPARATIONS | 0 ( 0.0%) | 0 ( 0.0%) | 1 ( 1.8%) | 1 ( 1.6%) |
| ..OTHER COLD PREPARATIONS | 0 ( 0.0%) | 0 ( 0.0%) | 0 ( 0.0%) | 2 ( 3.1%) |
| ..TONICS | 1 ( 1.6%) | 0 ( 0.0%) | 0 ( 0.0%) | 0 ( 0.0%) |
| ACE inhibitors and diuretics | 1 ( 1.6%) | 0 ( 0.0%) | 0 ( 0.0%) | 0 ( 0.0%) |
| ACE inhibitors,plain | 0 ( 0.0%) | 1 ( 1.5%) | 0 ( 0.0%) | 3 ( 4.7%) |
| Angiotensin II | 1 ( 1.6%) | 0 ( 0.0%) | 0 ( 0.0%) | 0 ( 0.0%) |
| antagonists and diuretics |  |  |  |  |
| Angiotensin II antagonists, plain | 1 ( 1.6%) | 2 ( 3.0%) | 1 ( 1.8%) | 0 ( 0.0%) |
| Beta blocking agents, selective | 1 ( 1.6%) | 0 ( 0.0%) | 0 ( 0.0%) | 0 ( 0.0%) |
| Biguanides | 1 ( 1.6%) | 0 ( 0.0%) | 0 ( 0.0%) | 0 ( 0.0%) |
| Calcium, combinations with vitamin D and/or other drugs | 1 ( 1.6%) | 1 ( 1.5%) | 0 ( 0.0%) | 1 ( 1.6%) |
|  |  |  |  |  |
| Code unknown | 0 ( 0.0%) | 4 ( 6.1%) | 1 ( 1.8%) | 0 ( 0.0%) |
| Corticosteroids | 0 ( 0.0%) | 0 ( 0.0%) | 1 ( 1.8%) | 0 ( 0.0%) |
| Glucocorticoids | 0 ( 0.0%) | 0 ( 0.0%) | 1 ( 1.8%) | 0 ( 0.0%) |
| HMG CoA reductase | 0 ( 0.0%) | 1 ( 1.5%) | 0 ( 0.0%) | 0 ( 0.0%) |
| inhibitors |  |  |  |  |
| Intrauterine | 1 ( 1.6%) | 0 ( 0.0%) | 1 ( 1.8%) | 0 ( 0.0%) |
| contraceptives |  |  |  |  |
| Iron bivalent, oral preparations | 0 ( 0.0%) | 0 ( 0.0%) | 0 ( 0.0%) | 1 ( 1.6%) |
| Iron in other combinations | 0 ( 0.0%) | 0 ( 0.0%) | 0 ( 0.0%) | 1 ( 1.6%) |
| Iron trivalent, oral preparations | 0 ( 0.0%) | 0 ( 0.0%) | 1 ( 1.8%) | 0 ( 0.0%) |
| Magnesium | 1 ( 1.6%) | 2 ( 3.0%) | 1 ( 1.8%) | 1 ( 1.6%) |
| Magnesium compounds | 0 ( 0.0%) | 0 ( 0.0%) | 0 ( 0.0%) | 1 ( 1.6%) |
| Multivitamins with minerals | 0 ( 0.0%) | 1 ( 1.5%) | 1 ( 1.8%) | 0 ( 0.0%) |
| Natural opium alkaloids | 0 ( 0.0%) | 0 ( 0.0%) | 0 ( 0.0%) | 1 ( 1.6%) |
| Other antidepressants | 1 ( 1.6%) | 0 ( 0.0%) | 0 ( 0.0%) | 0 ( 0.0%) |
| Other antiinflammatory and antirheumatic agents, non-steroids | 0 ( 0.0%) | 1 ( 1.5%) | 0 ( 0.0%) | 1 ( 1.6%) |
| Other antipsychotics | 1 ( 1.6%) | 0 ( 0.0%) | 0 ( 0.0%) | 0 ( 0.0%) |
| Other cardiac combination products | 0 ( 0.0%) | 0 ( 0.0%) | 0 ( 0.0%) | 1 ( 1.6%) |
| Other gynecologicals | 1 ( 1.6%) | 1 ( 1.5%) | 1 ( 1.8%) | 0 ( 0.0%) |
| Other lipid modifying agents | 0 ( 0.0%) | 2 ( 3.0%) | 1 ( 1.8%) | 0 ( 0.0%) |
| Other plain vitamin preparations | 1 ( 1.6%) | 1 ( 1.5%) | 0 ( 0.0%) | 0 ( 0.0%) |
| Platelet aggregation inhibitors excl. heparin | 0 ( 0.0%) | 1 ( 1.5%) | 0 ( 0.0%) | 0 ( 0.0%) |
| Potassium | 1 ( 1.6%) | 0 ( 0.0%) | 0 ( 0.0%) | 0 ( 0.0%) |
| Progestogens | 1 ( 1.6%) | 1 ( 1.5%) | 0 ( 0.0%) | 1 ( 1.6%) |
| Progestogens and estrogens, fixed combinations | 5 ( 8.2%) | 4 ( 6.1%) | 4 ( 7.0%) | 3 ( 4.7%) |
| Progestogens and estrogens, sequential preparations | 0 ( 0.0%) | 0 ( 0.0%) | 0 ( 0.0%) | 1 ( 1.6%) |
| Propionic acid derivatives | 0 ( 0.0%) | 1 ( 1.5%) | 0 ( 0.0%) | 0 ( 0.0%) |
| Proton pump inhibitors | 0 ( 0.0%) | 0 ( 0.0%) | 0 ( 0.0%) | 1 ( 1.6%) |
| Pyrazolones | 0 ( 0.0%) | 0 ( 0.0%) | 0 ( 0.0%) | 1 ( 1.6%) |
| Retinoids for treatment of acne | 1 ( 1.6%) | 0 ( 0.0%) | 0 ( 0.0%) | 0 ( 0.0%) |
| Selective serotonin reuptake inhibitors | 4 ( 6.6%) | 0 ( 0.0%) | 2 ( 3.5%) | 0 ( 0.0%) |
| Thyroid hormones | 2 ( 3.3%) | 2 ( 3.0%) | 1 ( 1.8%) | 2 ( 3.1%) |
| Vitamin A, plain | 0 ( 0.0%) | 0 ( 0.0%) | 0 ( 0.0%) | 1 ( 1.6%) |
| Vitamin B-complex, other combinations | 0 ( 0.0%) | 1 ( 1.5%) | 0 ( 0.0%) | 0 ( 0.0%) |
| Vitamin B12 (cyanocobalamin and analogues) | 1 ( 1.6%) | 2 ( 3.0%) | 0 ( 0.0%) | 1 ( 1.6%) |
| Vitamin D and analogues | 1 ( 1.6%) | 0 ( 0.0%) | 1 ( 1.8%) | 2 ( 3.1%) |
| Vitamins, other combinations | 0 ( 0.0%) | 1 ( 1.5%) | 0 ( 0.0%) | 0 ( 0.0%) |
| Zinc | 1 ( 1.6%) | 0 ( 0.0%) | 0 ( 0.0%) | 0 ( 0.0%) |
| **Begin before V1 and ongoing** |  |  |  |  |
|  |  |  |  |  |
| ..BETA BLOCKING AGENTS | 0 ( 0.0%) | 1 ( 1.5%) | 0 ( 0.0%) | 0 ( 0.0%) |
| ..GENERAL NUTRIENTS | 1 ( 1.6%) | 2 ( 3.0%) | 0 ( 0.0%) | 2 ( 3.1%) |
| ..HORMONAL CONTRACEPTIVES FOR SYSTEMIC USE | 0 ( 0.0%) | 0 ( 0.0%) | 1 ( 1.8%) | 0 ( 0.0%) |
| ..IRON PREPARATIONS | 0 ( 0.0%) | 0 ( 0.0%) | 0 ( 0.0%) | 1 ( 1.6%) |
| ..OTHER COLD PREPARATIONS | 0 ( 0.0%) | 0 ( 0.0%) | 0 ( 0.0%) | 1 ( 1.6%) |
| ..TONICS | 1 ( 1.6%) | 0 ( 0.0%) | 0 ( 0.0%) | 0 ( 0.0%) |
| ACE inhibitors and diuretics | 1 ( 1.6%) | 0 ( 0.0%) | 0 ( 0.0%) | 0 ( 0.0%) |
| ACE inhibitors, plain | 0 ( 0.0%) | 1 ( 1.5%) | 0 ( 0.0%) | 3 ( 4.7%) |
| Angiotensin II antagonists and diuretics | 1 ( 1.6%) | 0 ( 0.0%) | 0 ( 0.0%) | 0 ( 0.0%) |
| Angiotensin II antagonists, plain | 1 ( 1.6%) | 2 ( 3.0%) | 1 ( 1.8%) | 0 ( 0.0%) |
| Beta blocking agents, selective | 1 ( 1.6%) | 0 ( 0.0%) | 0 ( 0.0%) | 0 ( 0.0%) |
| Biguanides | 1 ( 1.6%) | 0 ( 0.0%) | 0 ( 0.0%) | 0 ( 0.0%) |
| Calcium, combinations with vitamin D and/or other drugs | 1 ( 1.6%) | 1 ( 1.5%) | 0 ( 0.0%) | 1 ( 1.6%) |
| Code unknown | 0 ( 0.0%) | 4 ( 6.1%) | 1 ( 1.8%) | 0 ( 0.0%) |
| Corticosteroids | 0 ( 0.0%) | 0 ( 0.0%) | 1 ( 1.8%) | 0 ( 0.0%) |
| HMG CoA reductase inhibitors | 0 ( 0.0%) | 1 ( 1.5%) | 0 ( 0.0%) | 0 ( 0.0%) |
| Intrauterine contraceptives | 1 ( 1.6%) | 0 ( 0.0%) | 1 ( 1.8%) | 0 ( 0.0%) |
| Iron bivalent, oral preparations | 0 ( 0.0%) | 0 ( 0.0%) | 0 ( 0.0%) | 1 ( 1.6%) |
| Iron in other combinations | 0 ( 0.0%) | 0 ( 0.0%) | 0 ( 0.0%) | 1 ( 1.6%) |
| Iron trivalent, oral preparations | 0 ( 0.0%) | 0 ( 0.0%) | 1 ( 1.8%) | 0 ( 0.0%) |
| Magnesium | 1 ( 1.6%) | 2 ( 3.0%) | 1 ( 1.8%) | 1 ( 1.6%) |
| Magnesium compounds | 0 ( 0.0%) | 0 ( 0.0%) | 0 ( 0.0%) | 1 ( 1.6%) |
| Multivitamins with minerals | 0 ( 0.0%) | 1 ( 1.5%) | 1 ( 1.8%) | 0 ( 0.0%) |
| Natural opium alkaloids | 0 ( 0.0%) | 0 ( 0.0%) | 0 ( 0.0%) | 1 ( 1.6%) |
| Other antidepressants | 1 ( 1.6%) | 0 ( 0.0%) | 0 ( 0.0%) | 0 ( 0.0%) |
| Other antiinflammatory and antirheumatic agents, non-steroids | 0 ( 0.0%) | 1 ( 1.5%) | 0 ( 0.0%) | 1 ( 1.6%) |
| Other antipsychotics | 1 ( 1.6%) | 0 ( 0.0%) | 0 ( 0.0%) | 0 ( 0.0%) |
| Other cardiac combination products | 0 ( 0.0%) | 0 ( 0.0%) | 0 ( 0.0%) | 1 ( 1.6%) |
| Other gynecologicals | 1 ( 1.6%) | 1 ( 1.5%) | 1 ( 1.8%) | 0 ( 0.0%) |
| Other lipid modifying agents | 0 ( 0.0%) | 2 ( 3.0%) | 1 ( 1.8%) | 0 ( 0.0%) |
| Other plain vitamin preparations | 1 ( 1.6%) | 1 ( 1.5%) | 0 ( 0.0%) | 0 ( 0.0%) |
| Platelet aggregation inhibitors excl. heparin | 0 ( 0.0%) | 1 ( 1.5%) | 0 ( 0.0%) | 0 ( 0.0%) |
| Potassium | 1 ( 1.6%) | 0 ( 0.0%) | 0 ( 0.0%) | 0 ( 0.0%) |
| Progestogens | 1 ( 1.6%) | 1 ( 1.5%) | 0 ( 0.0%) | 1 ( 1.6%) |
| Progestogens and estrogens, fixed combinations | 5 ( 8.2%) | 4 ( 6.1%) | 4 ( 7.0%) | 3 ( 4.7%) |
| Progestogens and estrogens, sequential preparations | 0 ( 0.0%) | 0 ( 0.0%) | 0 ( 0.0%) | 1 ( 1.6%) |
| Propionic acid derivatives | 0 ( 0.0%) | 1 ( 1.5%) | 0 ( 0.0%) | 0 ( 0.0%) |
| Proton pump inhibitors | 0 ( 0.0%) | 0 ( 0.0%) | 0 ( 0.0%) | 1 ( 1.6%) |
| Pyrazolones | 0 ( 0.0%) | 0 ( 0.0%) | 0 ( 0.0%) | 1 ( 1.6%) |
| Retinoids for treatment of acne | 1 ( 1.6%) | 0 ( 0.0%) | 0 ( 0.0%) | 0 ( 0.0%) |
| Selective serotonin reuptake inhibitors | 4 ( 6.6%) | 0 ( 0.0%) | 2 ( 3.5%) | 0 ( 0.0%) |
| Thyroid hormones | 2 ( 3.3%) | 2 ( 3.0%) | 1 ( 1.8%) | 2 ( 3.1%) |
| Vitamin A, plain | 0 ( 0.0%) | 0 ( 0.0%) | 0 ( 0.0%) | 1 ( 1.6%) |
| Vitamin B-complex, other combinations | 0 ( 0.0%) | 1 ( 1.5%) | 0 ( 0.0%) | 0 ( 0.0%) |
| Vitamin B12 (cyanocobalamin and analogues) | 1 ( 1.6%) | 2 ( 3.0%) | 0 ( 0.0%) | 1 ( 1.6%) |
| Vitamin D and analogues | 1 ( 1.6%) | 0 ( 0.0%) | 1 ( 1.8%) | 2 ( 3.1%) |
| Vitamins, other combinations | 0 ( 0.0%) | 1 ( 1.5%) | 0 ( 0.0%) | 0 ( 0.0%) |
| Zinc | 1 ( 1.6%) | 0 ( 0.0%) | 0 ( 0.0%) | 0 ( 0.0%) |
| **Begin after V1** |  |  |  |  |
| ..TONICS | 0 ( 0.0%) | 0 ( 0.0%) | 1 ( 1.8%) | 0 ( 0.0%) |
| Antiseptics | 1 ( 1.6%) | 0 ( 0.0%) | 0 ( 0.0%) | 0 ( 0.0%) |
| Antivirals | 0 ( 0.0%) | 0 ( 0.0%) | 0 ( 0.0%) | 1 ( 1.6%) |
| Code unknown | 0 ( 0.0%) | 0 ( 0.0%) | 0 ( 0.0%) | 1 ( 1.6%) |
| Estren derivatives | 1 ( 1.6%) | 0 ( 0.0%) | 0 ( 0.0%) | 0 ( 0.0%) |
| Propionic acid derivatives | 0 ( 0.0%) | 1 ( 1.5%) | 0 ( 0.0%) | 0 ( 0.0%) |
| Thyroid hormones | 0 ( 0.0%) | 0 ( 0.0%) | 0 ( 0.0%) | 1 ( 1.6%) |
| Vitamin D analogues | 0 ( 0.0%) | 0 ( 0.0%) | 1 ( 1.8%) | 1 ( 1.6%) |

Each ATC 4 level is counted only once per patient Level 2 or 3 is displayed if coding at lower level was not possible

**1.2 Supplemental figures**

Figure S1: Remission to absent or mild symptoms


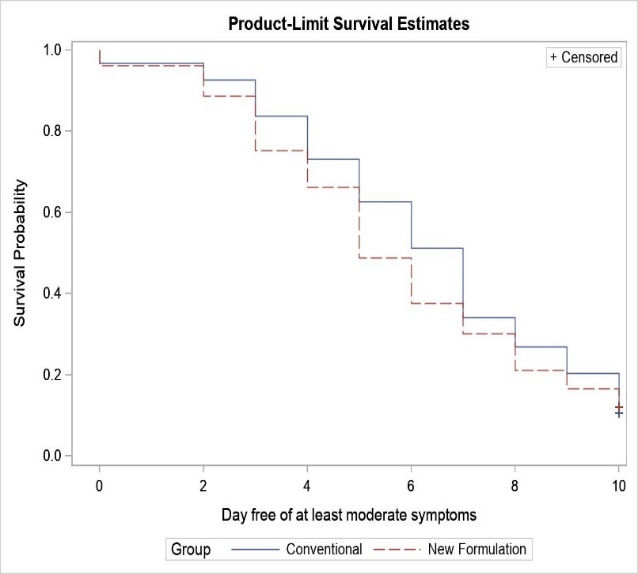
a. b.


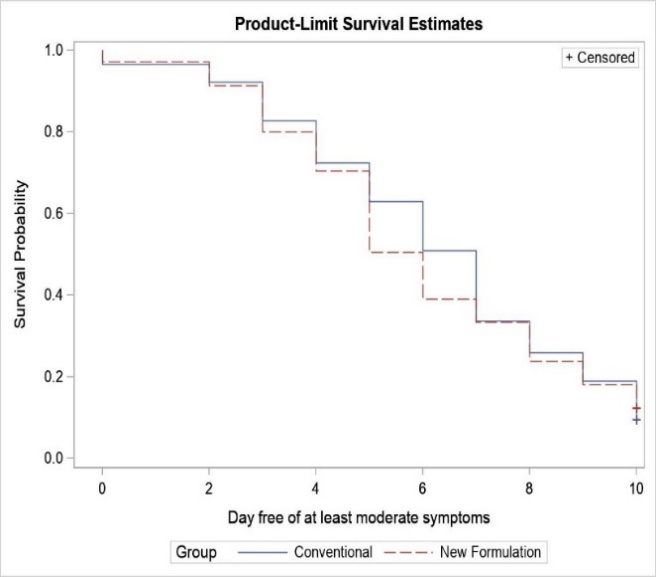


Probability of Recovery

Probability of Recovery

Legend figure S1: Remission of first (a) and all (b) infection episodes, comparison of recovery times of new (dashed line, A + B) versus conventional (solid line, C + D) (p=0.36 and p=0.08) formulations until day 10; y-axis: probability of recovery; x-axis: day free of at least moderate symptoms; Kaplan-Meier analysis.

Figure S2: Remission of first infection episode, cross comparisons between single formulations

1.
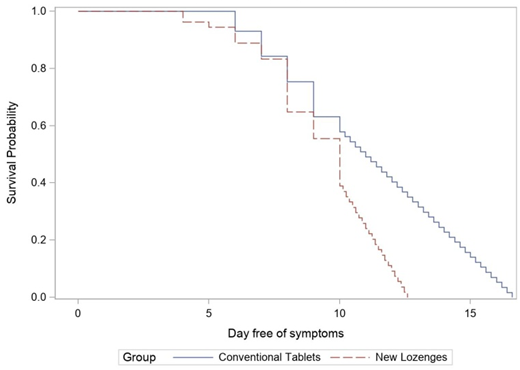

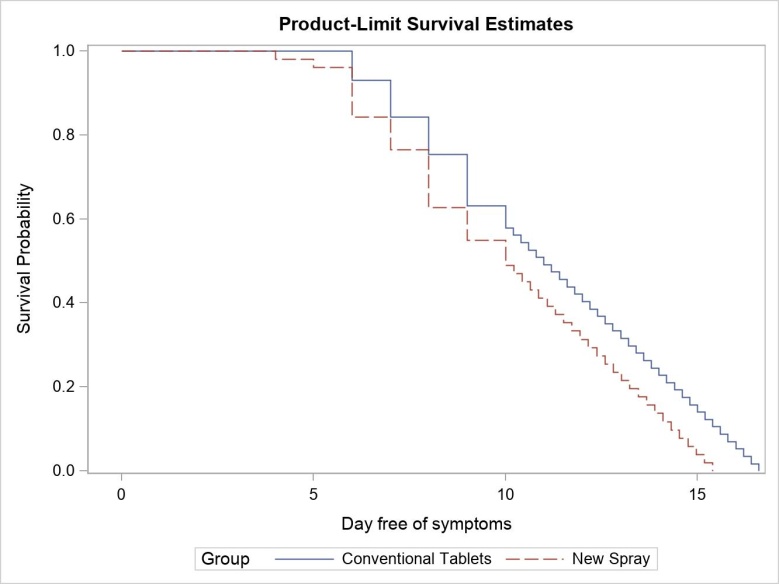
b.

Probability of Recovery

Probability of Recovery


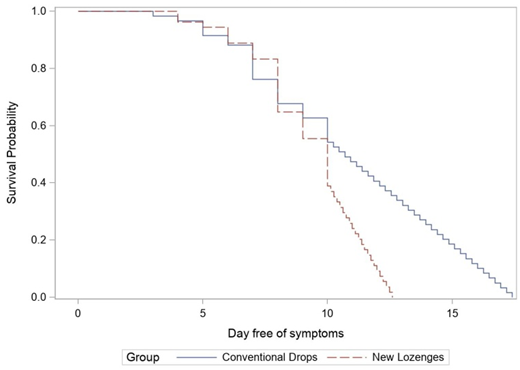

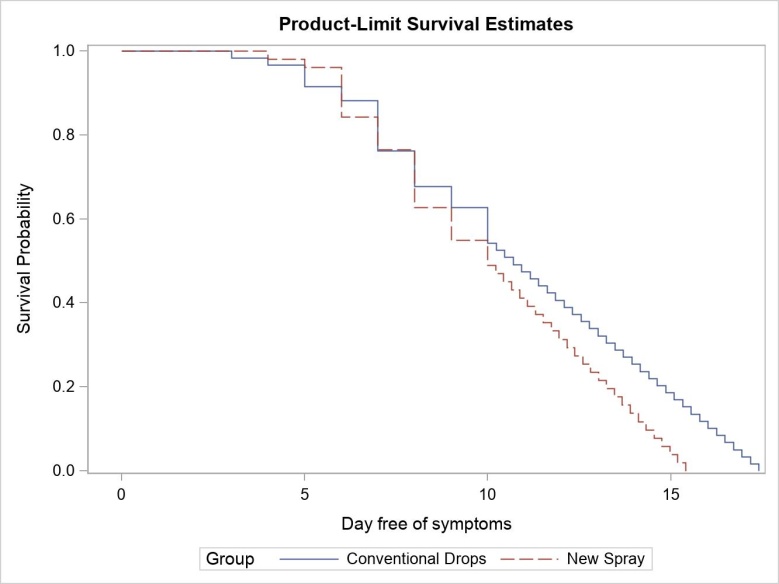
c. d.

Probability of Recovery

Probability of Recovery

Probability of Recovery

Legend figure S2 a-d: Comparison of recovery times of (a) lozenges (dashed line) versus conventional tablets (solid line) (p<0.001), (b) spray (dashed line) versus conventional tablets (solid line) (p=0.09), (c) spray (dashed line) versus conventional drops (solid line) (p=0.25), (d) lozenges (dashed line) versus conventional drops (solid line) (p=0.01), extrapolated beyond day 10 to absent symptoms; y-axis: probability of recovery; x-axis: day free of symptoms; Kaplan-Meier analysis.

Figure S3: Remission of first infection episode with remission defined as mild or absent symptoms


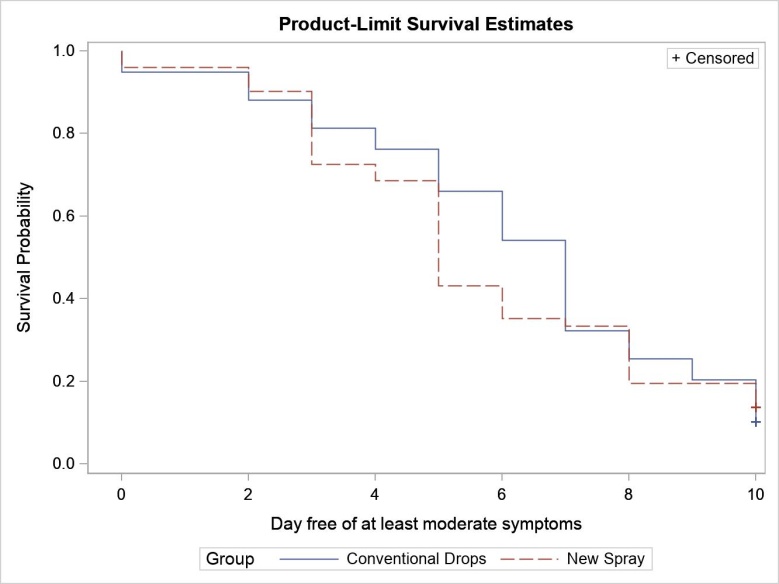


Probability of Recovery

b.


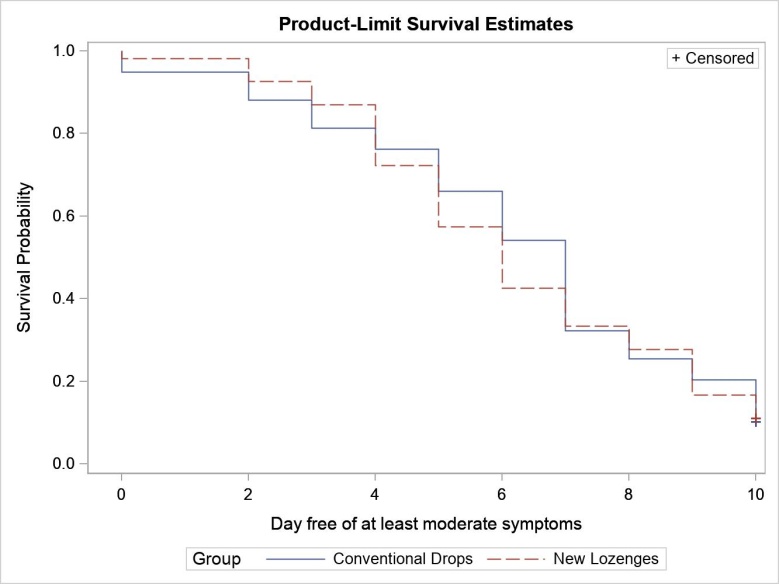


Probability of Recovery

Legend figure S3: Comparison of recovery times of (a) spray (dashed line) versus conventional drops (solid line) and (b) lozenges (dashed line) versus conventional tables (solid line) until day 10; y-axis: probability of recovery; x-axis: day free of at least moderate symptoms; Kaplan-Meier analysis

Figure S4: Remission of all infection episodes with remission defined as mild or absent symptoms


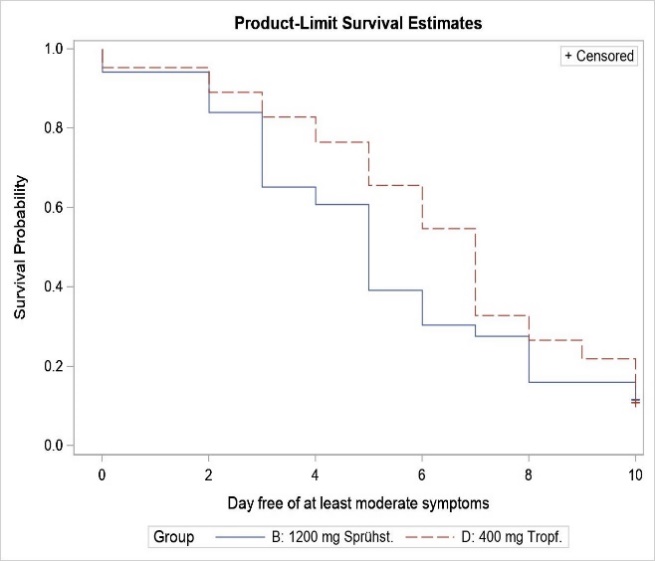


Probability of recovery

b.


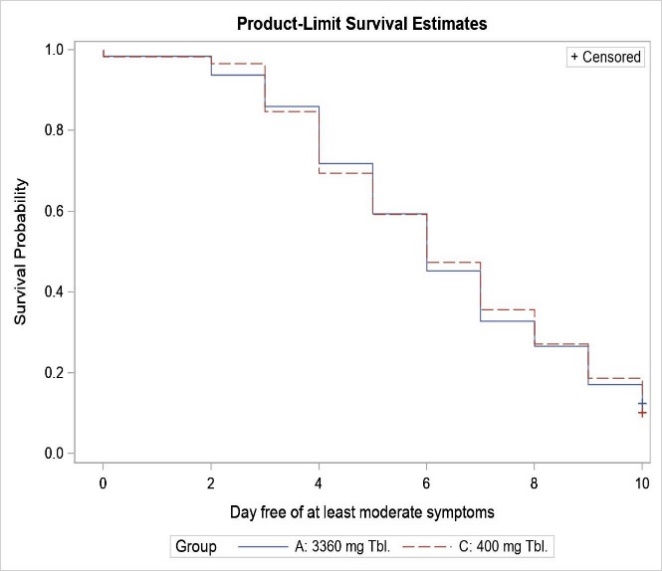


Probability of recovery

Legend figure S4: Comparison of recovery times of (a) spray (solid line) versus conventional drops (dashed line) (p=0.12) and (b) lozenges (solid line) versus conventional tables (dashed line) (p=0.96) until day 10 (y-axis: probability of recovery, x-axis: day free of at least moderate symptoms).

Figure S5 a-h

Development of Single Symptom Runny nose, Congested nose, Sneezing, Cough, Shivering, Sore throat, Feeling unwell, Headache, Muscle ache.

a.
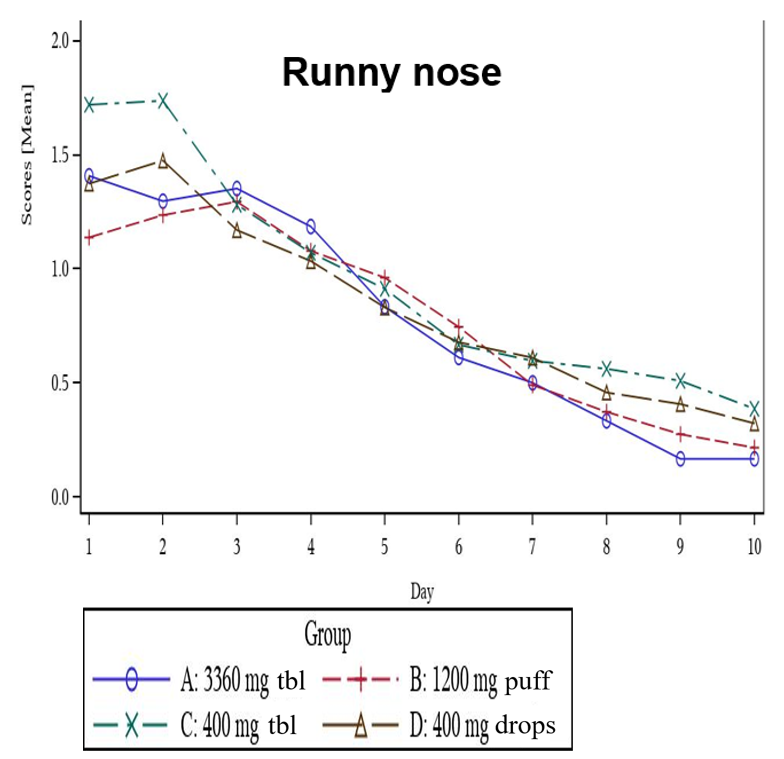


b.
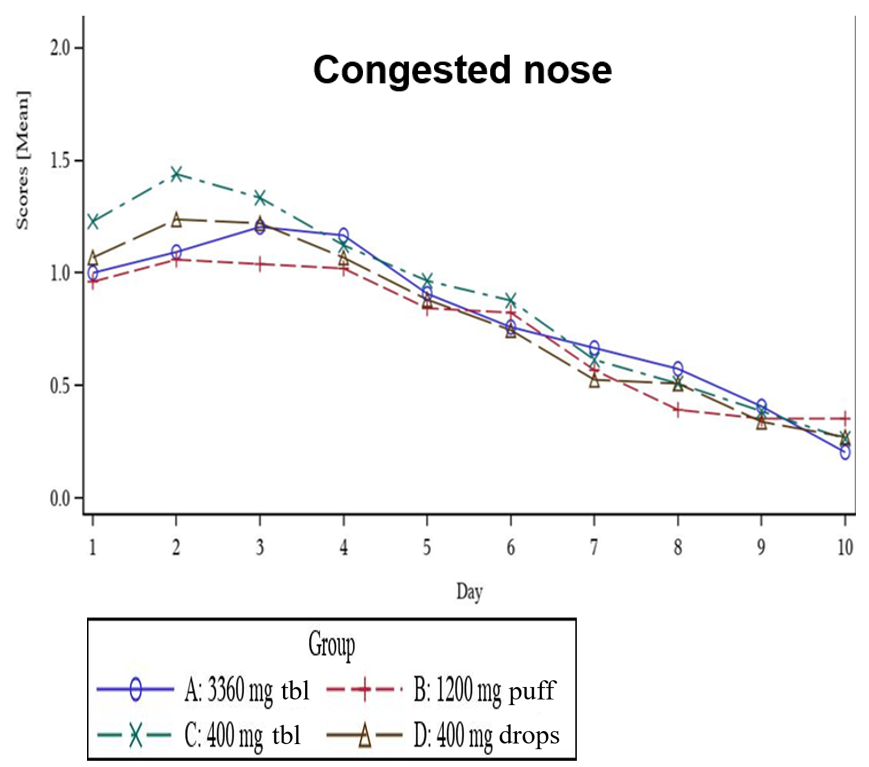


c.
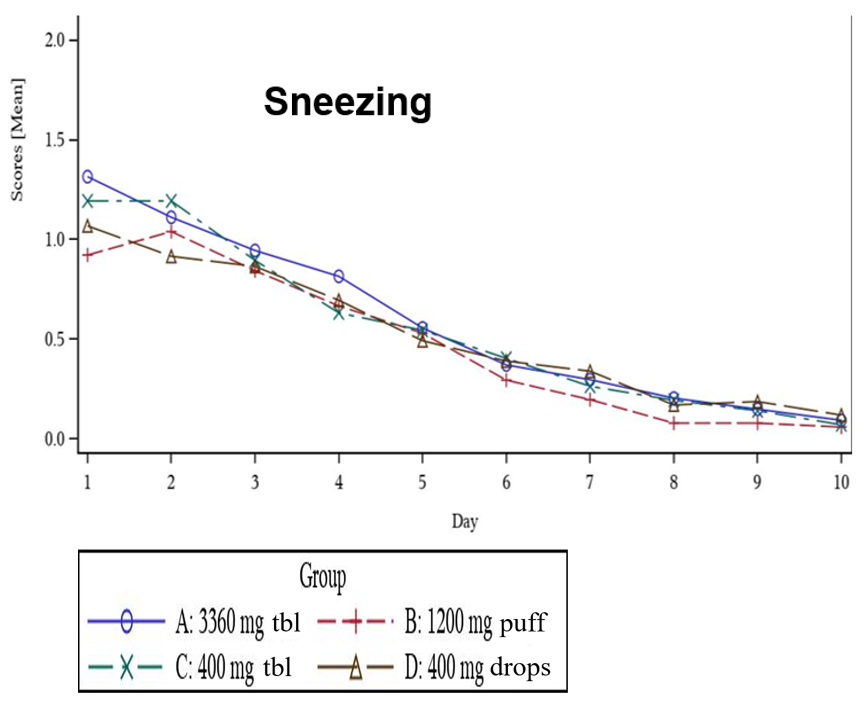


d.
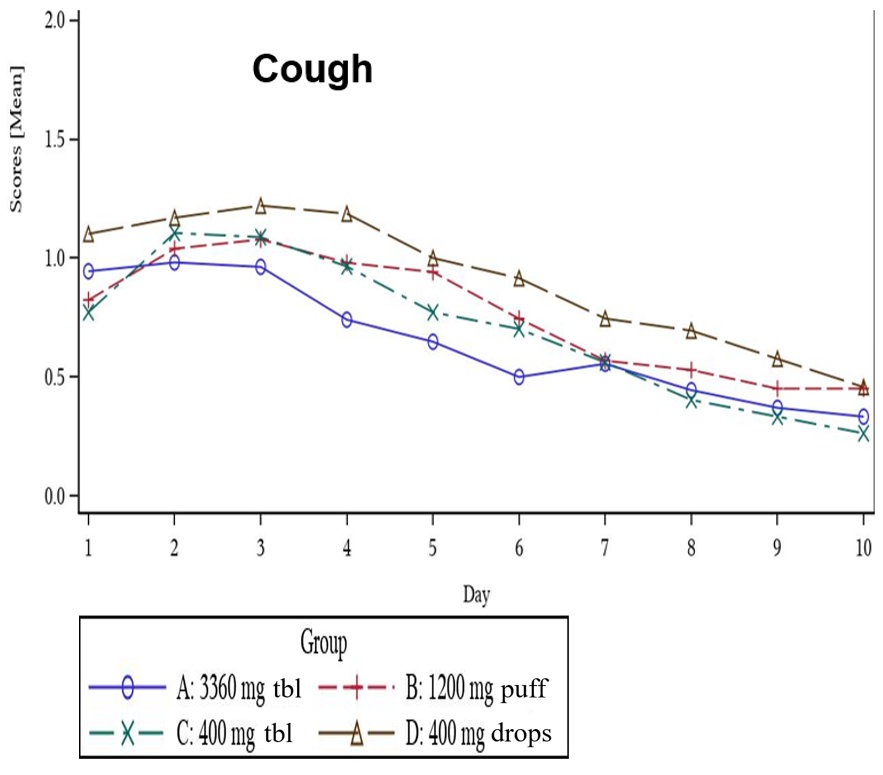


e.
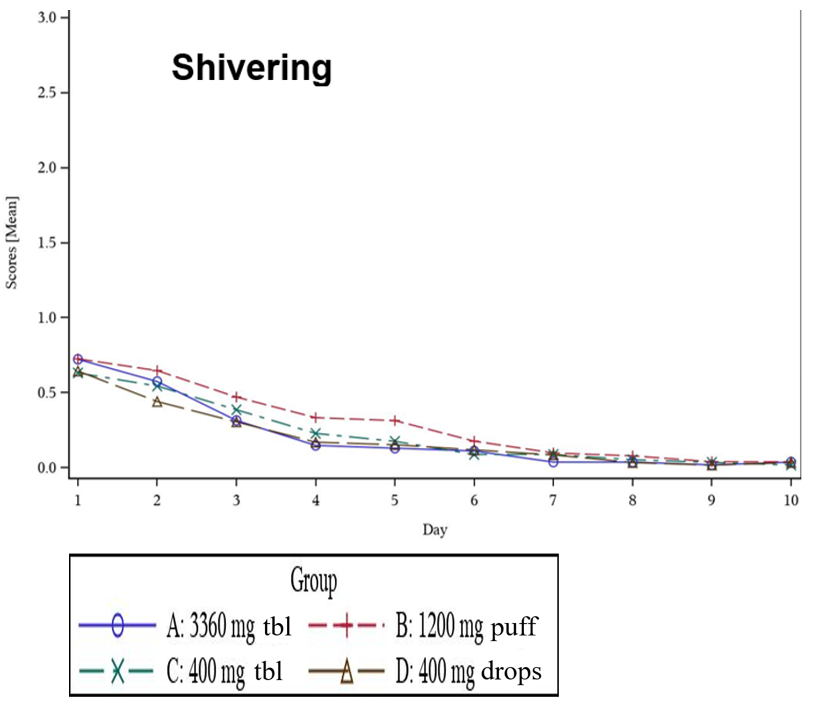


f.
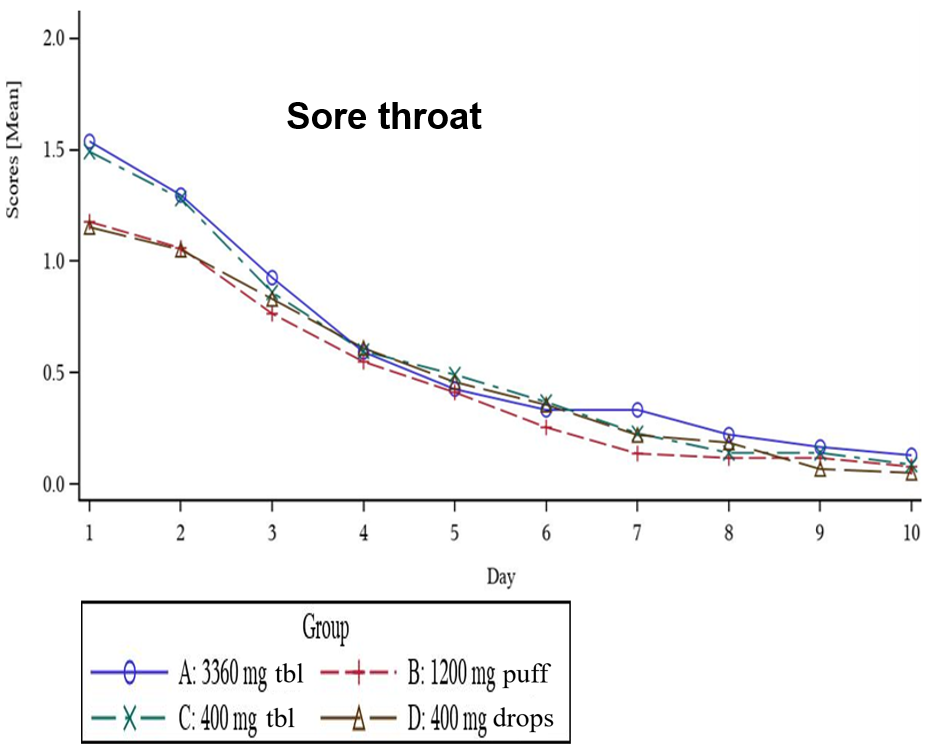


g.
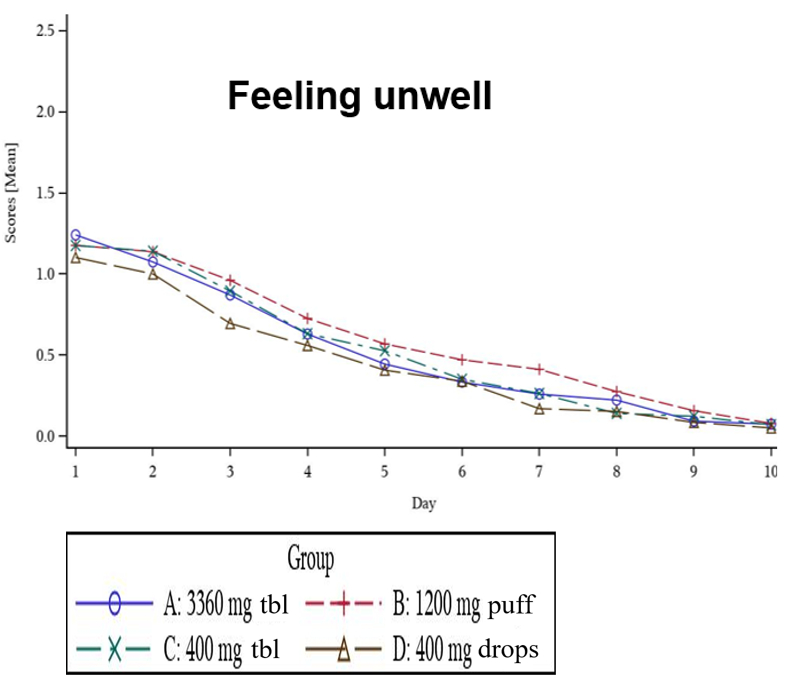


h.
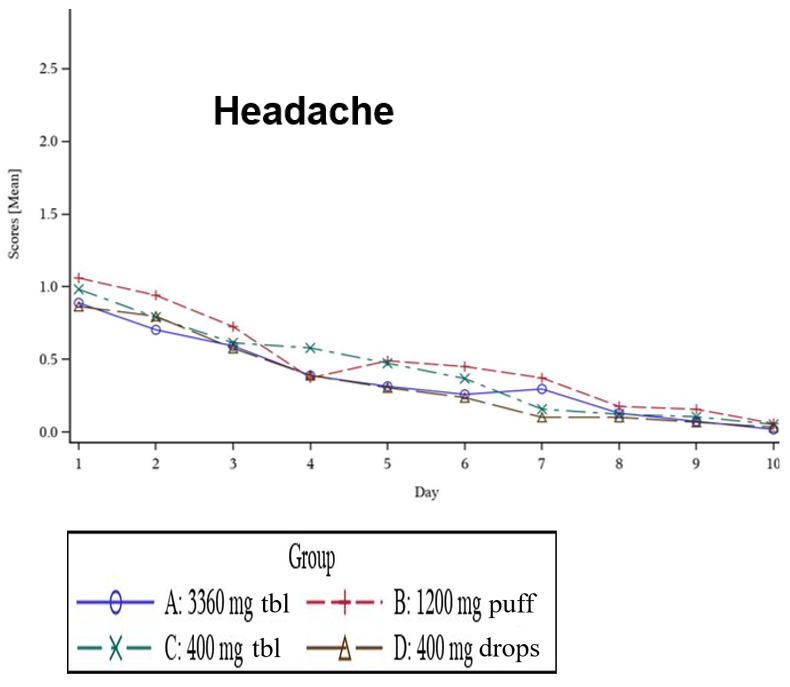


i.
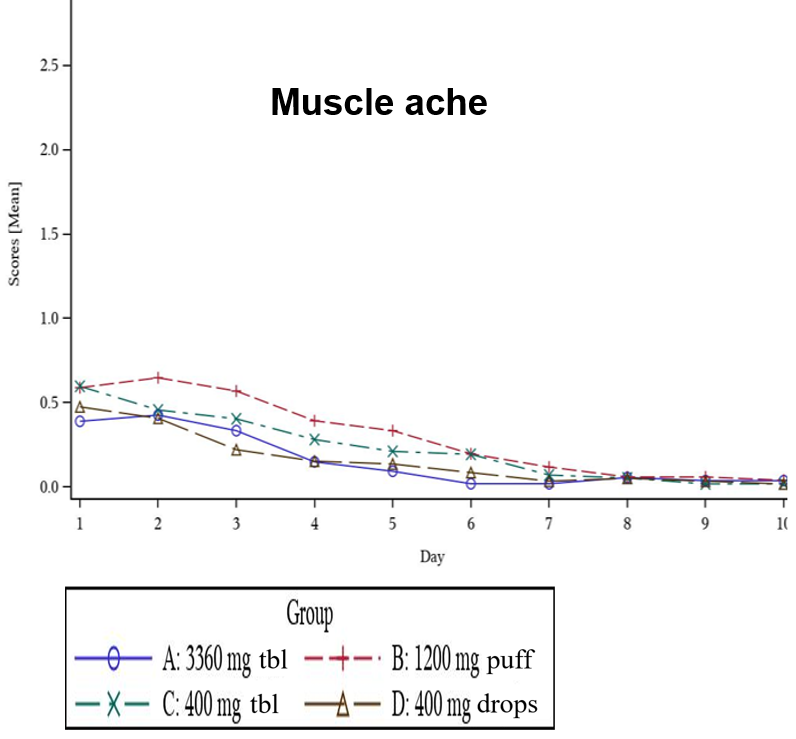

Supplement: Supplementary file 1 [file Data_Sheet_1.docx]
